# Distinct prion conformers from brain and peripheral tissues of gene-targeted mice produce convergent CWD strain properties

**DOI:** 10.1371/journal.ppat.1014303

**Published:** 2026-06-04

**Authors:** Joseph P. DeFranco, Zoe N. Atkinson, Jenna Crowell, Xutong Shi, Sehun Kim, Julianna L. Sun, Sarah J. Kane, Glenn C. Telling

**Affiliations:** 1 Prion Research Center, Department of Microbiology, Immunology, and Pathology, Colorado State University, Fort Collins, Colorado, United States of America; 2 Program in Cell and Molecular Biology, Colorado State University, Fort Collins, Colorado, United States of America; National Institutes of Health, NIAID, UNITED STATES OF AMERICA

## Abstract

Prions are unique infectious agents because different conformational properties of their constituent proteins are responsible for the manifestation of distinct strains. While prion replication in humans with sporadic Creutzfeldt-Jakob disease and cattle with bovine spongiform encephalopathy is primarily limited to the central nervous system (CNS), a seminal feature of chronic wasting disease (CWD) in cervids is high infectious titers in peripheral tissues, including skeletal muscles and the lymphoreticular system. Although extensive studies have assessed prion properties in the CNS, our understanding of prions in peripheral tissues remains limited. Here, we compared the strain properties of CWD prions in peripheral and CNS tissues of gene-targeted (Gt) mice. Our studies reveal differences in the biochemical and conformational properties of CWD prions, as well as the prion levels, between peripheral and CNS tissues. While this finding suggested that these tissues harbored distinct CWD prions, transmissions of muscle, spleen, and brain homogenates to Gt mice by the intraperitoneal route produced convergent strain properties. Importantly, transmission of these tissues by the intracerebral route resulted in different disease phenotypes than intraperitoneal inoculations. Additionally, while prion infection of CWD-susceptible cells revealed different titers in muscle, spleen, and brain tissues, the conformational properties of the resulting de novo prions were indistinguishable. While our findings support a role for tissue-specific cofactors that affect the biochemical and conformational properties of prions, they also show that these parameters do not solely dictate disease outcomes and that additional factors, particularly the route of inoculation, exert a more pronounced influence on strain outcomes.

## Author summary

The contamination of the food chain by bovine spongiform encephalopathy prions, which caused disease in animals and humans, exemplifies the lethal consequences of interspecies prion transmissions. Because prion diseases also threaten wildlife ecology, it is essential to understand prion replication within the host organism and how strain differences influence the likelihood of transmission within and between species. Chronic wasting disease (CWD), a burgeoning prion epidemic of cervids, presents with high prion titers in peripheral tissues and bodily fluids, which most likely explains its irrevocable spread across North America. Here, we investigated CWD prions in the brains, spleens, and skeletal muscles of gene-targeted mice expressing the cervid prion protein. While we found that the biochemical and conformational properties, as well as the levels of CWD prions, in the brain and peripheral tissues were distinct, transmissions to gene-targeted mice produced convergent strain outcomes. These findings further validate the use of gene-targeted mice for modeling prion diseases and provide insights into the biological processes underlying strain evolution.

## Introduction

Prion diseases are a group of fatal neurological disorders that affect animals and humans [1]. While sporadic Creutzfeldt-Jakob disease (sCJD) prions in humans and bovine spongiform encephalopathy (BSE) prions in cattle primarily replicate within the central nervous system (CNS), scrapie prions in small ruminants and chronic wasting disease (CWD) prions in cervids propagate in peripheral tissues like skeletal muscles and the lymphoreticular system (LRS) [2–5]. The CWD epidemic is unique because it is the only prion disease affecting captive as well as free-ranging animals, with diseased cervids documented in North America, South Korea, and Northern Europe [6–9]. The contagious irrevocable spread of CWD in North America is thought to be related to the high titers of the infectious agent in peripheral tissues, which is excreted or shed into the environment and subsequently spread throughout the ecosystem [10]. Because these diseases threaten public health, agriculture, and wildlife ecology, it is essential to understand the dynamics of the infectious agent, its replication within the host organism, and the potential for intra- and interspecies transmission.

While it is well established that the conformational change of the cellular prion protein, PrP^C^, to the pathogenic isoform (PrP^Sc^) is the molecular basis of prion propagation, the ability or inability of prions to replicate in different tissues remains poorly understood. We have previously described the production of gene-targeted (Gt) mice that express cervid PrP from the endogenous murine *Prnp* locus [11–13]. Because North American elk express glutamate (E) at codon 226, whereas deer, moose, and reindeer express glutamine (Q), we developed Gt mice expressing these two sequences, termed GtE and GtQ, respectively [11]. These Gt mice are fully susceptible to CWD prions by peripheral routes of exposure with attendant prion accumulation in LRS tissue, which makes them a suitable model for studying peripheral pathogenesis. Despite the importance of prions in the periphery, most assessments in experimental settings have focused on prions in the brain and on their subsequent transmission by the intracerebral (ic) route of inoculation in transgenic (Tg) mice [14]. Consequently, the properties of prions within peripheral tissues and their transmission properties to susceptible models are not well understood.

Prion strains are considered to be populations of PrP^Sc^ conformers with specific biochemical and conformational properties that produce defined disease phenotypes [15–22]. Strain properties affect time to disease onset, clinical signs, the spread of PrP^Sc^ throughout the organism, the distribution of neuropathology, and infectious titer in CNS tissue at the terminal stage of disease. Our previous findings indicate that the route of inoculation of identical CWD isolates can influence the resulting prion conformational properties and disease outcomes [15]. Other groups have also found tissue-specific effects, such as more permissive prion replication in the LRS than in brain tissue following interspecies transmission [23]. These data collectively led to our hypothesis that selective pressures within the microenvironments of peripheral tissues and the CNS influence prion replication and strain phenotypes [15]. Here, we investigated the properties of prions in the brains and peripheral tissues of CWD-infected Gt mice and monitored the resulting strain properties following ic and intraperitoneal (ip) challenges in additional GtE and GtQ mice, as well as infection of susceptible cultured mammalian cells expressing elk PrP.

## Results

### Distinct conformational and biochemical properties of CWD prions from brain, spleen, and muscle tissues of Gt mice

**Fig 1:**
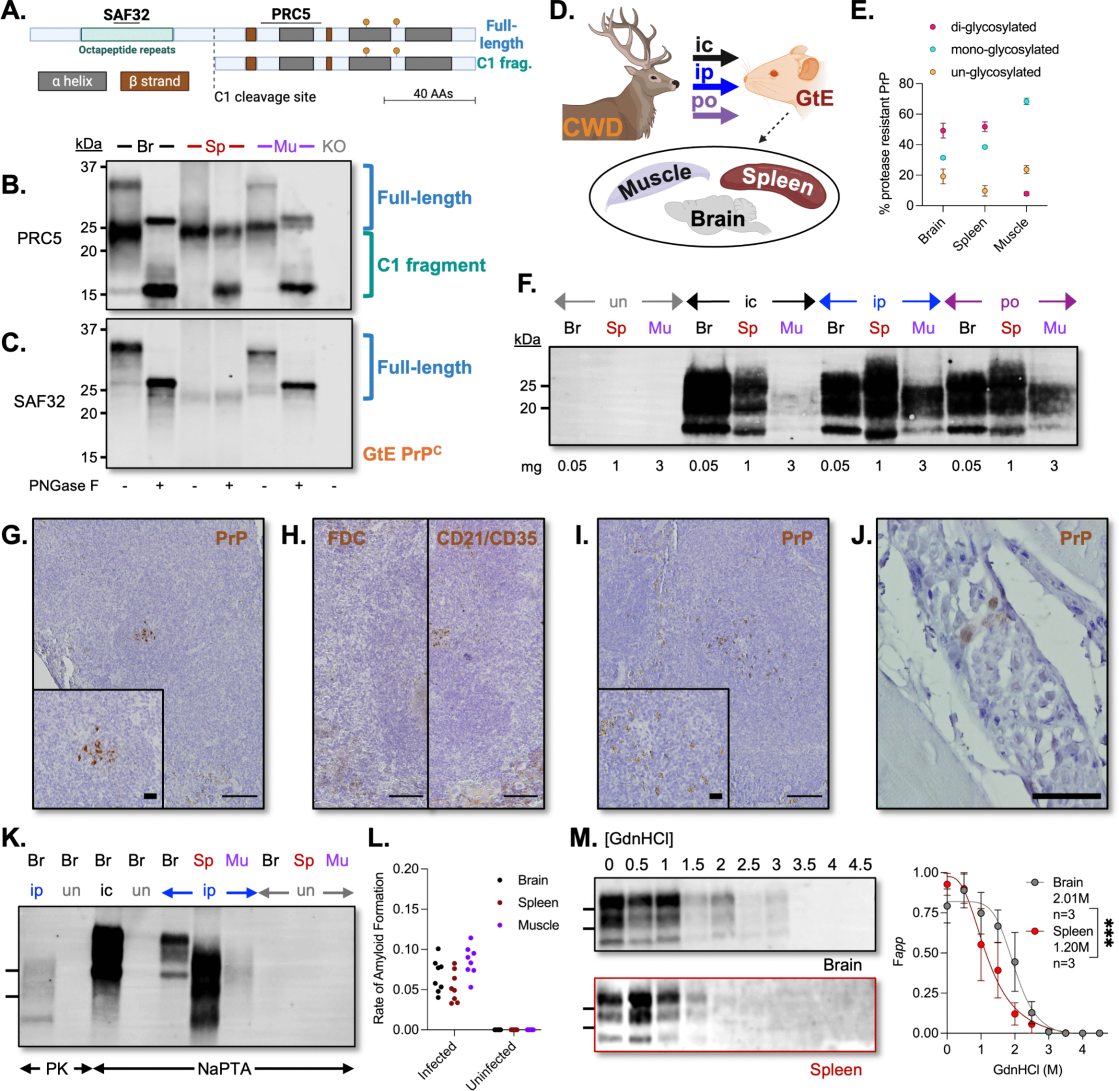
The biochemical and conformational properties of PrP^C^ and CWD prions in brain, spleen, and muscle tissue of GtE mice. **A.,** schematic of mature PrP^C^ and location of endogenous proteolytic C1 cleavage event which produces a full-length species and the C1 fragment (frag.). N-linked glycosylation sites are depicted as yellow circles. The epitopes for the anti-PrP antibodies SAF32 and PRC5 are shown. Created in BioRender. Defranco, **J.** (2026) https://BioRender.com/tomd10m. **B. – C.,** western blotting analyses of different tissues from uninfected GtE mice. For brain (Br) tissue, 10 µg of protein was loaded. For spleen (Sp) tissue, 50 µg of protein was loaded. For muscle (Mu) tissue, 100 µg of protein was loaded. For a control, 10 µg of *Prnp*^*-/-*^ (KO) Br tissue was loaded. Tissues were untreated (-) or treated with PNGase F (+) prior to immunoblotting. Membranes were probed with either **B.,** mAb PRC5 or **C.,** mAb SAF32 and the positions of 37, 25, 20, and 15 kDa molecular weight markers are shown. **D.,** schematic of North American elk CWD prion transmissions to GtE mice by the intracerebral (ic), intraperitoneal (ip), and *per os* (po) routes of inoculation. Brain, spleen, and muscle tissues were collected at the terminal stage of prion disease. Created in BioRender. Defranco, **J.** (2026) https://BioRender.com/tomd10m. **E.,** ratio of CWD PrP^Sc^ glycoforms from diseased GtE brain, spleen, and muscle tissue following all routes of inoculation from Fig 1F. Data points represent the mean (±SD) of the relative proportion from three animals of di-glycosylated (red), mono-glycosylated (blue), and un-glycosylated (gold) CWD PrP^Sc^. Locations of glycoform quantifications are described in S2 Fig. **F.,** western blotting of proteinase K-treated brain (Br), spleen (Sp), or muscle (Mu) homogenates. Mice were either uninfected (un), ic-inoculated, ip-inoculated, or po-inoculated with elk CWD prions. The amounts of protein loaded (mg) for each tissue homogenates are shown. **G. – H.,** immunohistochemical analyses of serial spleen tissue sections from an ip-inoculated diseased GtE mouse; **G.,** was probed with anti-PrP Fab d18; **H.,** was probed with anti-follicular dendritic cell (FDC) Ab (clone FDC-M1) or anti-CD21/CD35 ab (clone 7G6) as indicated. **I.,** immunohistochemical analyses of spleen tissue from an uninfected GtE mouse probed with anti-PrP Fab d18. **J.** immunohistochemical analyses of muscle spindle from an ip-inoculated diseased GtE mouse probed with anti-PrP Fab d18. For **G. – J.,** thin scale bars, 50 µM; bold scale bars, 20 µM. **K.,** tissue homogenates following Pronase E treatment and sodium phosphotungstic acid (NaPTA) precipitation or proteinase K (PK) digestion. Tissues were collected from uninfected or diseased GtE mouse following ic or ip inoculation with elk CWD prions. For PK-treated samples, 50 µg of protein was loaded. For NaPTA precipitated samples, 100 µg of protein was loaded for brain samples, 2 mg for spleen samples, and 4 mg for muscle samples. **L.,** RT-QuIC of infected or uninfected tissue homogenates following NaPTA precipitation and Pronase E treatment. Data is expressed as the Rate of Amyloid Formation, which is the inverse of the time to threshold. **M.,** representative western blots of infected brain and spleen homogenate subjected to increasing concentrations of guanidine hydrochloride (GdnHCl) followed by proteinase K (PK) treatment. Quantification of conformational stability analyses of brain and spleen homogenates is displayed as average fractional apparent signal (*Fapp*) ± SEM after PK digestion and is plotted as a function of GdnHCl concentration **(M)**. Sigmoidal dose-response curves were plotted using a four-parameter algorithm. The concentrations of GdnHCl to produce half-maximal denaturation of prions [GdnHCl_1/2_] are also shown. Significance was determined by comparing the best fit line between replicates (***, *P* ≤ 0.001). For western blots in panels **F., K., and M.**, the positions of 25 and 20 kDa molecular weight markers are shown and blots were probed with mAb PRC5.

GtE mice express elk PrP^C^ in the brain and peripheral tissues at levels equivalent to mouse PrP^C^ expressed in wild-type mice [11,13]. Western blotting demonstrated that PrP^C^ levels were the highest in the brain tissue, followed by spleen and muscle (S1 Fig). This finding supports previous analyses in wild-type and knock-in mice and higher mammals [12,24–27]. In order to assess endoproteolytic processing of elk PrP^C^ in different tissues of GtE mice, we enzymatically removed N-linked glycans with PNGase F and probed Western blots with mAb PRC5 or SAF32 (Fig 1A-1C). The epitopes of mAbs PRC5 and SAF32 reside in the globular domain and octapeptide repeats of PrP^C^, respectively. Accordingly, while PRC5 recognizes full-length PrP and C1 [28,29], SAF32 recognizes only full-length PrP. Probing de-glycosylated PrP^C^ with SAF32 revealed that full-length PrP^C^ from brain and muscle migrated at ~ 27 kDa, while full-length PrP^C^ from spleen migrated at ~ 24 kDa. Also, while full-length PrP^C^ in brain and muscle was predominately fully glycosylated, full-length PrP^C^ from spleen was almost entirely un-glycosylated. These findings build upon previous exploratory analyses of PrP^C^ in different tissues [23–26]. The presence of significant amounts of de-glycosylated C1 following PNGase F treatment also revealed that the majority of C1 in these tissues was N-glycosylated. These findings reveal that elk PrP^C^ is differentially glycosylated and processed in spleen, muscle, and brain tissues of GtE mice.

Following successful transmission of CWD prions to GtE mice expressing cervid PrP by the ic, ip, and *per os* (po) routes of inoculation (S1 Table) [11,15], we collected brain, spleen, and skeletal muscle tissues (Fig 1D). Western blotting analyses of proteinase K (PK)-resistant PrP^Sc^ revealed the accumulation of CWD prions in the brain, spleen, and muscle of GtE mice (Fig 1E, 1F and S2 Fig). Compared with infected brain tissue, spleen and muscle required >10-fold higher total protein levels to detect PK-resistant PrP^Sc^ by western blotting, suggesting lower prion levels in peripheral tissues. We found that these levels of PK-resistant PrP^Sc^ were proportional to the levels of PrP^C^ in the tissues of GtE mice (S1 Fig). Immunohistochemistry (IHC) analyses confirmed that ic and peripheral challenges produced prion deposition in the spleens of diseased GtE and GtQ mice (Fig 1G and S3 Fig). There were no discernible differences in prion deposition in splenic tissues following different routes of inoculation or cervid PrP genotypes. Because our previous work demonstrated that peripheral routes of inoculation authentically recapitulate the natural strain properties of native CWD and that there are no strain differences between peripherally inoculated GtE and GtQ mice [15], we conducted the majority of further analyses on tissues collected from peripherally inoculated GtE mice. We probed with a follicular dendritic cell (FDC)-specific marker, FDC-M1, and a CD21/CD35 antibody to confirm the location of germinal centers in GtE mice (Fig 1H and S4 Fig). We observed anti-PrP immunostaining in the germinal centers of ip-inoculated GtE splenic tissue (Fig 1G and S3 Fig), which is consistent with previous findings from CWD-infected cervids [30] and laboratory-adapted rodent prions [31–34]. We also found non-disease-specific PrP staining in the red pulp and germinal centers of uninfected GtE spleens that was absent in *Prnp*^*-/-*^ mice (Fig 1H, 1I; S3 and S4 Figs). We attribute this observation to the high expression of PrP^C^ by splenic mast cells as previously reported in mice and deer [35,36]. Immunohistochemical analyses also confirmed CWD prion accumulation in muscle spindles of GtE mice, which is consistent with previous reports [37] (Fig 1J and S5 Fig). Collectively, these analyses demonstrate that CWD prions accumulate in the spleen and muscle tissues of diseased Gt mice.

We compared the biochemical and conformational properties of CWD prions derived from brain, spleen, and muscle tissues by several different approaches. Western blot analyses revealed that the glycoform ratios of PK-resistant PrP^Sc^ differ across tissues (Fig 1E, 1F and S2 Fig). Brain and spleen CWD prions from Gt mice are primarily di-glycosylated, which is a characteristic of North American CWD prions [38,39]. Interestingly, CWD prions derived from muscle were different because they lacked high levels of di-glycosylated PrP^Sc^ (Fig 1E and 1F). Because the glycosylation patterns and endoproteolytic processes of PrP^C^ from the brain and muscle of GtE mice were indistinguishable (Fig 1B and 1C), these data suggest that other factors control prion glycosylation in these tissues. Additionally, in comparison to brain-derived prions, we observed slightly elevated mono-glycosylated PrP^Sc^ and decreased levels of un-glycosylated PrP^Sc^ within spleen CWD prions of GtE mice. Following selective protein precipitation using sodium phosphotungstic acid (NaPTA) and digestion with pronase E of homogenates from ip-inoculated GtE mice, we observed different electrophoretic mobilities of the purified PrP preparations from brain and spleen tissue (Fig 1K). The molecular weight of splenic prions following NaPTA precipitation corresponded to truncated PrP of PK-resistant PrP^Sc^ from brain tissue, which was between 17–27 kDa. The molecular weight of purified brain prions was similar to full-length PrP at 25–35 kDa (Fig 1K and S6 Fig). Whereas we readily detected PrP^Sc^ in muscle tissue following PK treatment and ultracentrifugation, levels of muscle prions were much lower after NaPTA precipitation. Despite CNS and peripheral tissues producing different molecular weights following NaPTA precipitation, real-time quaking-induced conversion (RT-QuIC) confirmed amyloid seeding activity in preparations from all three tissues (Fig 1L). We also measured the conformational properties of brain and spleen prions by assessing their transitions from PK-resistant conformations to PK-susceptible states following treatment with increasing concentrations of guanidine hydrochloride (GdnHCl) [40]. Prions from brain tissue required elevated levels of GdnHCl of 2.01 M to produce half-maximal denaturation ([GdnHCl_1/2_]) compared to prions derived from spleen tissue which only required 1.20 M (Fig 1M). Taken together, these data suggest that the biochemical and conformational properties of CWD prions produced in the brain, spleen, and muscle are different.

### Brain, spleen, and muscle tissues of CWD-infected GtE mice harbor different prion titers

**Fig 2:**
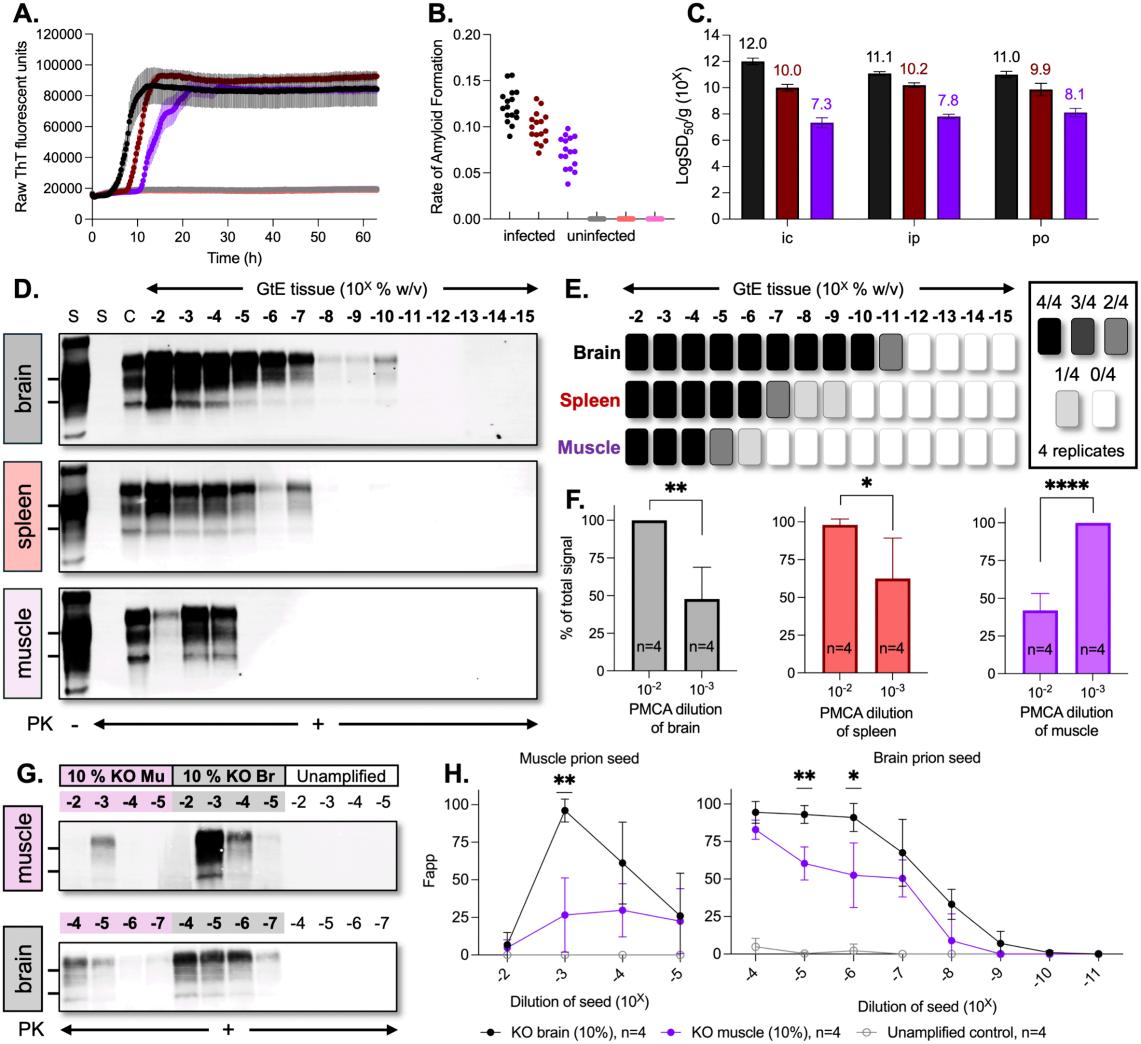
*In vitro* amplification efficiency of prions from brain, spleen, or muscle tissue of CWD-infected GtE mice A. – C, seeding activity following RT-QuIC of ip-inoculated brain (black), spleen (dark red), muscle (purple) tissue or uninfected brain (gray), spleen (orange), or muscle (pink) tissue. In each condition, four different tissues were assessed independently four times: **A.,** average (±SEM) raw fluorescent values of thioflavin T (ThT) following a 62.5-hour **(h)** RT-QuIC assay; **B.,** data is expressed as the Rate of Amyloid Formation, which is the inverse of the time to threshold. **C.,** graphical representation of prion titer (±SD) computed by the Spearman-Kärber method by determining the seeding dose (SD) in which there are 50% positive reactions. For each inoculation group, three GtE mice were analyzed, and the individual titer values are summarized in S2 Table. **D.** representative western blots showing the PMCA products following proteinase K (PK) treatment as indicated. Reactions were seeded with brain, spleen, or muscle homogenate from diseased GtE mice ip-inoculated with CWD prions as indicated. Non-amplified brain homogenate controls are shown: S, uninfected GtE brain homogenate; C, elk CWD prion-infected GtE mouse brain homogenate. PMCA products of serial dilutions 10^-2^ to 10^-15^% (w/v) of CWD seed are shown in bold. **E.,** quantification of results from four PMCA experiments in GtE brain homogenates with seeds from four different animals. For each condition, products following PMCA reactions in which the seed ranges from 10^-2^ to 10^-15^% (w/v). **F.,** quantification of western blots lanes of 10^-2^ and 10^-3^ dilutions from PMCA experiments using brain, spleen, or muscle seeds. Total PK-resistant PrP^Sc^ band signal was computed by densitometric analysis of western blots. For each replicate, signals of the higher lane was arbitrarily set to be 100% signal, and the other lane was expressed as a fraction of the signal. Average fractions of total signals are plotted (±SD). **G.,** PMCA experiments in which muscle or brain seeds were added to a substrate mixture containing either 10% (w/v) of muscle or brain tissue from a *Prnp*^-/-^ mouse. A schematic for the experimental design is shown in S8 Fig. Serial dilutions of the seed from 10^-2^ to 10^-7^% (w/v) are shown. Reactions subjected to PMCA are bolded and all samples were treated with PK prior to western blotting. **H.,** quantification (±SD) for PMCA experiments for muscle and brain seeds. Lanes containing reactions with 10% (w/v) of KO muscle homogenate subjected to PMCA are purple, and lanes containing reactions with 10% (w/v) of KO brain homogenate subjected to PMCA are black. Lanes containing unamplified seeds used for PMCA are gray. In all cases, significance was assessed using Student’s unpaired t-test (****, *P* ≤ 0.0001; **, *P* ≤ 0.01; *, *P* ≤ 0.05). For western blots in **D.** and **G.**, the positions of 25 and 20 kDa molecular weight markers are shown and blots were probed with mAb PRC5.

To address whether the levels of PK-resistant PrP^Sc^ in CWD-infected brain, spleen, and muscle tissues (Fig 1B) correlated with variable levels of prion replication, we used RT-QuIC to measure the prion seeding activity in different tissues. We confirmed that crude preparations of all three tissues from ip-inoculated GtE mice induced amyloid seeding using recombinant Syrian hamster PrP (90–231) as substrate (Fig 2A and 2B). We assessed prion titers in the three tissues of ic-, ip-, and po-inoculated mice using the Spearman-Kärber method following RT-QuIC end-point dilution [41]. We found the highest levels of amyloid seeding in brain, followed by spleen and muscle tissues (Fig 2C and S2 Table), which correlated with their levels of PK-resistant PrP^Sc^ (Fig 1B). The different titers in brain, spleen, and muscle homogenates were consistently observed in ic-, ip-, and po-inoculated GtE mice. We also found ~ 10-fold higher levels of seeding activity in the brains of ic-inoculated mice compared with their peripherally inoculated counterparts (Fig 2C and S2 Table), which is consistent with our previous findings using the cervid prion cell assay (CPCA) and 7–5 ELISA [15]. In contrast to the variable titers in diseased brains, we found no differences in the prion seeding activity of peripheral tissues following different routes of inoculation (Fig 2C and S2 Table). In summary, while brain homogenates from CWD-infected GtE mice contain the highest levels of seeding activity, crude spleen and muscle homogenates also produce robust amyloid seeding activity.

We also investigated the *in vitro* prion amplification capacity of prions from brain, spleen, and muscle homogenates to convert elk PrP^C^ from GtE mouse brain tissue using protein misfolding cyclic amplification (PMCA). Similar to the RT-QuIC results, we found that brain homogenate from ip-inoculated GtE mice resulted in the highest prion amplification, followed by spleen and muscle seeds (Fig 2D, 2E and S7 Fig). Interestingly, we observed consistently poorer amplification in the lowest dilution of PMCA products following muscle seeding compared to reactions initiated with either brain or spleen inoculum (Fig 2F). To test whether this was a component of the prion seed or the residual muscle tissue present in the inoculum, we used infected brain and muscle homogenates to seed substrates containing either 10% brain or muscle tissue from a *Prnp*^*-/-*^ mouse [42,43] (S8 Fig). In both cases, PMCA amplification was less efficient in the substrate containing the muscle tissue from a *Prnp*^*-/-*^ mouse (Fig 2G and 2H). These data suggest that there is an intrinsic component of muscle tissue, independent of PrP^C^, that causes an inhibitory effect in a cell-free prion conversion system. Our demonstrations of lower levels of prion replication in the peripheral tissues of diseased GtE mice correlate with previous findings in CWD-infected cervids [3,44].

### Indistinguishable strain properties and disease outcome following inoculation with CWD prions from brain, spleen, and muscle tissues

**Fig 3:**
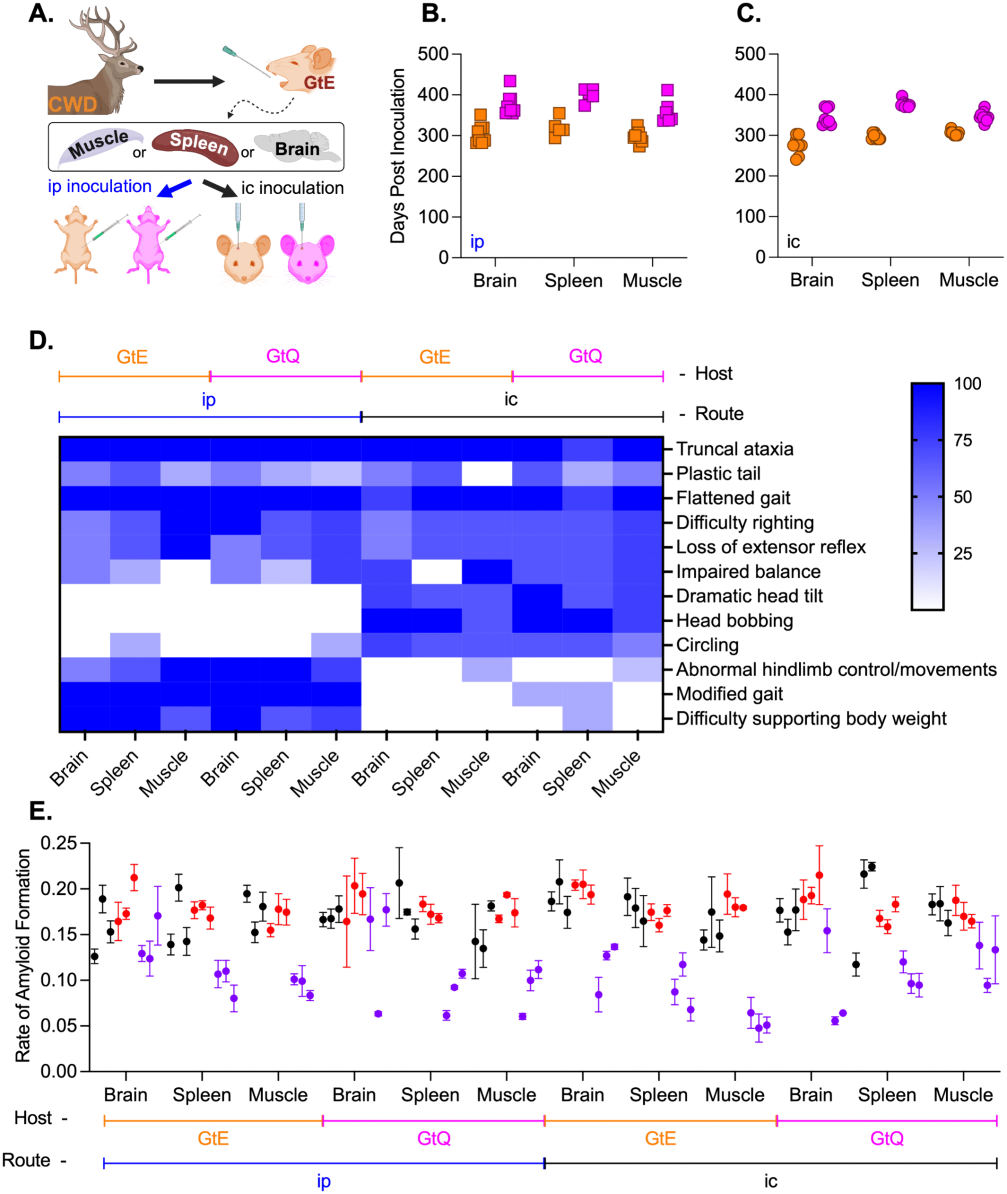
Transmission of CWD-infected GtE muscle, spleen, and brain tissue to additional Gt mice. **A.,** schematic of passage history of CWD prions to Gt mice. Following oral gavage challenge with elk CWD prions, infected brain, spleen, and muscle tissues from GtE mice were homogenized and serially transmitted to GtE or GtQ mice by the intraperitoneal (ip) or intracerebral (ic) routes of inoculation. Created in BioRender. Defranco, **J.** (2026) https://BioRender.com/tomd10m. **B. – C.,** prion disease transmissions to diseased GtE (orange) and GtQ (magenta) mice. Average times to disease onset are summarized in Table 1. Mice were inoculated either with brain, spleen, or muscle homogenates by the **B.,** ip or **C.,** ic routes of inoculation. Each data point represents a diseased mouse. **D.,** heat map assessment of prion disease-specific clinical signs at terminal stage of disease. Three to four mice were analyzed and the presence or absence of consistent and obvious clinical signs were plotted with dark blue as the most prevalent, light blue as low prevalence, and white as absent (legend indicates the percentage of mice). **E.,** RT-QuIC using brain (black), spleen (red), and muscle (purple) homogenates following ip or ic inoculation with CWD-infected brain, spleen or muscle tissue. For each condition, three biological replicates were assessed four times. Data is expressed as the Rate of Amyloid Formation (±SEM), which is the inverse of the time to threshold.

Our findings of distinct conformational and biochemical properties, variable prion levels, and tissue-specific replication environments suggested that different prion strains accumulated in CNS and peripheral tissues. To address this further, we performed serial transmissions of brain, spleen, and muscle homogenates from a clinically sick GtE mouse, orally challenged with native elk CWD prions, by the ip and ic routes to additional GtE and GtQ mice (Fig 3A). Challenges of Gt mice with brain, spleen, and muscle tissue homogenates produced disease in all cases (Fig 3B-3E and Table 1). Average times to disease onset ranged from 276 to 306 d in ic-challenged GtE mice and from 346 to 376 d in ic-challenged GtQ mice. Mean incubation periods ranged from 297 to 320 d in ip-challenged GtE mice and from 359 to 400 d in ip-challenged GtQ mice. Three-way ANOVA showed only modest influences of the route of inoculation and whether inocula originated from brain, spleen or muscle, but a significant effect of host genotype (S3 Table). Indeed, in accordance with our previous observations following transmissions of natural CWD isolates from North America and South Korea [11,13,15,45,46], in all cases, disease was significantly more rapid in GtE than GtQ mice (Table 1). We evaluated disease-specific clinical signs in Gt mice inoculated with prions from brain, spleen, or muscle homogenates by the ip or ic routes (Fig 3D). Whereas all transmissions resulted in truncal ataxia, flattened gait, difficulty righting, loss of extensor reflex, impaired balance, and occasional plastic tail, the majority of ic-inoculated mice showed pronounced head tilt, head bobbing, and circling. In contrast, the majority of ip-inoculated mice exhibited abnormal hindlimb control and movements, modified gait, and had difficulty supporting their body weight at the terminal stage of disease. We conclude that different routes of inoculation produce distinct clinical signs.

**Table 1:** Transmission of CWD-infected brain, spleen, and muscle homogenate to GtE and GtQ mice. Time to disease onset (incubation time) is expressed as the average time, in days, at which inoculated mice first developed ultimately progressive signs of neurological disease. Variance is expressed as ± standard error of the mean (SEM). Prion disease was confirmed by real-time quaking-induced conversion (RT-QuIC), neurohistopathological analyses, and/or by western immunoblotting of CNS prions. Mice dying of intercurrent illnesses prior to prion disease onset were excluded from these calculations. 𝚫 GtE – GtQ, differences in mean incubation times of transmissions of individual tissues during passages to GtE or GtQ mice determined by the Mantel-Cox test.

Tissue	Intraperitoneal			Intracerebral		
	GtE	GtQ	Δ GtE – GtQ	GtE	GtQ	Δ GtE – GtQ
Brain	305 ± 9 (8/8)	376 ± 6 (9/9)	19% (*P* < 0.0001)	276 ± 8 (8/8)	346 ± 7 (9/10)	21% (*P* < 0.0001)
Spleen	320 ± 10 (5/5)	400 ± 6 (6/6)	20% (*P* < 0.001)	297 ± 2 (9/9)	376 ± 2 (10/10)	22% (*P* < 0.0001)
Muscle	297 ± 5 (9/9)	359 ± 12 (6/6)	17% (*P* < 0.01)	306 ± 2 (10/10)	346 ± 4 (10/10)	12% (*P* < 0.0001)

**Fig 4:**
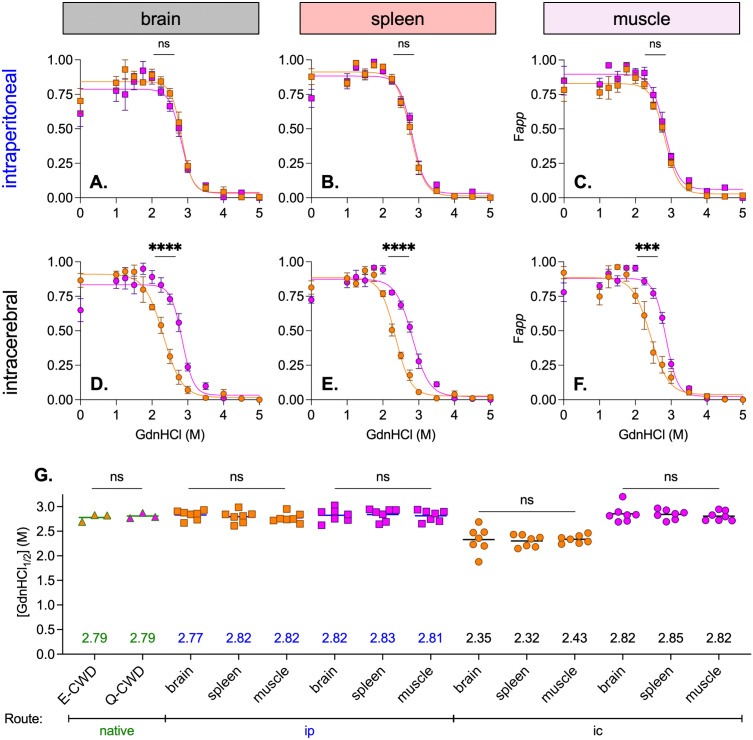
Conformational stabilities of prions from the CNS of Gt mice following intraperitoneal or intracerebral transmissions with CWD-infected brain, spleen or muscle tissue. **A. – F.,** responses of prions to increasing concentrations of GdnHCl. In all cases, GtE mice are orange and GtQ mice are magenta. Average fractional apparent signal (F*app*) ± SEM after PK digestion is plotted as a function of GdnHCl concentration **(M)**. Sigmoidal dose-response curves were plotted using a four-parameter algorithm. The concentrations of GdnHCl to produce half-maximal denaturation of prions [GdnHCl_1/2_] are shown in panel **G.** for each condition. In all cases, samples with elk PrP genotype (E226) are orange and deer PrP genotype (Q226) are magenta, and three mice were independently analyzed two to three times. Prions from the brains of Gt mice following **A.,** intraperitoneal (ip) inoculation with CWD-infected brain homogenate; **B.,** ip inoculation with CWD-infected spleen homogenate; **C.,** ip inoculation with CWD-infected muscle homogenate; **D.,** intracerebral (ic) inoculation with CWD-infected brain homogenate; **E.,** ic inoculation with CWD-infected spleen homogenate; **F.,** ic inoculation with CWD-infected muscle homogenate. **G.,** Graphical representation of [GdnHCl_1/2_] values of CWD prions from native elk and deer and Gt mice following ip- or ic-passage. Responses of native elk and deer CWD prions to increasing concentrations of GdnHCl shown in S9 Fig. For all cases, significance was determined by comparing [GdnHCl_1/2_] values between replicates (****, *P* ≤ 0.0001; ns, *P* > 0.05).

To address whether the biochemical and conformational differences of PrP^Sc^ observed in brain, spleen, and muscle tissues of GtE-infected mice (Fig 1) were maintained upon transmission, we assessed the conformational properties of prions in the brains of diseased GtE and GtQ mice. In concordance with our previous results [15], native elk and deer CWD isolates had the same [GdnHCl_1/2_] of 2.79 M (Fig 4G and S9 Fig), and this prion conformation was retained upon oral transmission to GtE mice [15]. Upon ip inoculation of infected brain, spleen, or muscle prions into GtE and GtQ mice, we observed no difference in the denaturation profile (Fig 4A-4C), with [GdnHCl_1/2_] values ranging from 2.77 to 2.83 M (Fig 4G). In contrast to the indistinguishable conformational profiles of native CWD and its peripherally propagated derivatives in Gt mice, ic inoculation of native CWD results in the divergent denaturation profiles and distinct [GdnHCl_1/2_] values [15]. Upon ic transmission of muscle, spleen, and brain tissue from an orally inoculated mouse, we also observed divergent denaturation profiles (Fig 4D-4F), with distinct [GdnHCl_1/2_] values ranging from 2.32 to 2.43 M for GtE prions and 2.82 to 2.85 M for GtQ prions (Fig 4G). While we observed conformational differences within prions resident in CNS and peripheral tissues (Fig 1), transmission of prions from brain, spleen, and muscle homogenates produced indistinguishable prion conformational properties within the brains of diseased mice (Fig 4G). Collectively, these data indicate that the conformational properties of CWD prions are influenced by the route of inoculation but are unaffected by inoculation with different tissue sources.

**Fig 5:**
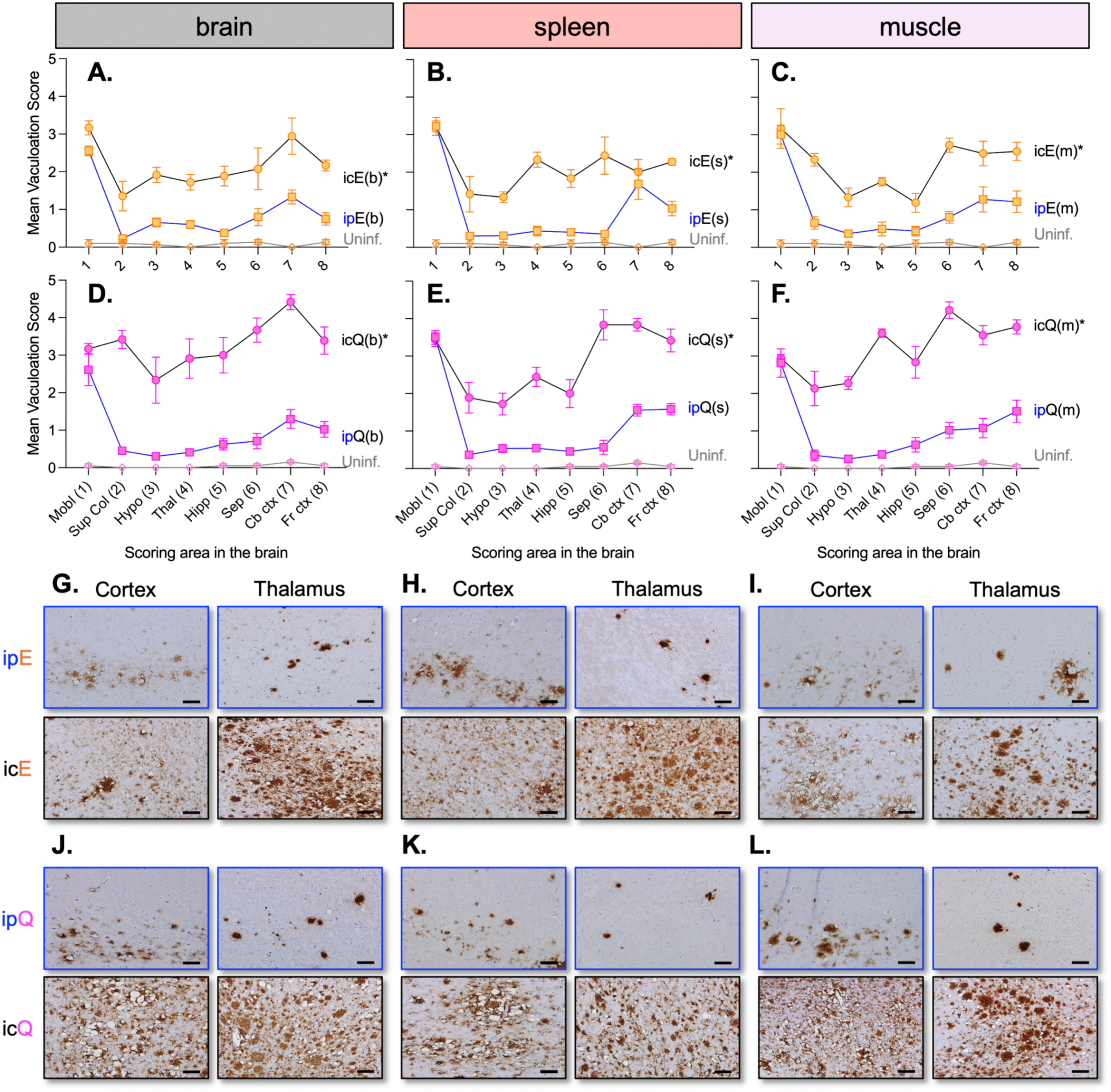
Neuropathological outcomes following transmission of CWD-infected muscle, spleen, or brain tissue to GtE and GtQ mice. **A – F.,** assessment of vacuolar degeneration in the following regions: 1, medulla oblongata (Mobl); 2, superior colliculus (Sup Col); 3, hypothalamus (Hypo); 4, thalamus (Thal); 5, hippocampus (Hipp); 6, septal area (Sep); 7, cerebral cortex at the level of the thalamus (Cb ctx); 8, frontal cerebral cortex (Fr ctx). Severity of vacuolation was scored on a scale of zero to five. Data points represent mean scores ± SEM for at least three mice per group. Orange symbols, GtE mice; magenta symbols, GtQ mice. Mice are uninfected (Uninf., diamonds) or inoculated by the intracerebral (ic) (circles) or intraperitoneal (ip) (squares) routes. *, ic-inoculated Gt mice exhibited asymmetric spongiform degeneration in the forebrain and the scores for both hemispheres were averaged before plotting. **G. – L.,** immunohistochemical analyses of disease-associated prion protein in either the cerebral cortex or thalamus of ip- or ic-inoculated GtE and GtQ mice. Scale bars, 100 µM. Tissues were probed with Fab D18. All images were sections of the right hemisphere, which was ipsilateral to the side of inoculation for ic-inoculated mice. **A.** and **G.,** GtE mice were challenged with CWD-infected muscle homogenate; **B.** and **H.,** GtE mice were challenged with CWD-infected spleen homogenate; **C.** and **I.,** GtE mice were challenged with CWD-infected brain homogenate. **D.** and **J.,** GtQ mice were challenged with CWD-infected muscle homogenate; **E.** and **K.,** GtQ mice were challenged with CWD-infected spleen homogenate; **F.** and **L.,** GtQ mice were challenged with CWD-infected brain homogenate.

Additional assessments of disease phenotype confirmed that the route of inoculation was a more significant strain determinant than the tissue source used for the inoculum. While the location of spongiform degeneration of ip-inoculated GtE and GtQ mice was invariable, with higher severity in the hindbrain, ic-inoculated mice harbored higher levels of vacuolation and modest differences between GtE and GtQ mice in the superior colliculus, thalamus, hippocampus, and septal area (Fig 5A-5F). While we did observe greater lesion variability in the brains of ic-inoculated mice across inoculation groups with different tissue inocula, these differences were not significant. Immunohistochemical analyses of disease-associated PrP demonstrated that the cortex and thalamic regions of ip-inoculated GtE and GtQ mice contained limited CWD prion deposition composed of coarse aggregates (Fig 5G-5I). In contrast, the brains of ic-inoculated GtE and GtQ mice contained high levels of PrP^Sc^ deposition in these regions, which comprised a mix of coarse and punctate aggregates (Fig 5J-5L). There were no discernible differences in the location or morphology of prion deposition in diseased Gt mice inoculated with brain, spleen, or muscle tissues. Overall, these results suggest that the route of inoculation plays a larger role in the neuropathology phenotype than the inoculum tissue source in CWD-infected mice.

**Fig 6:**
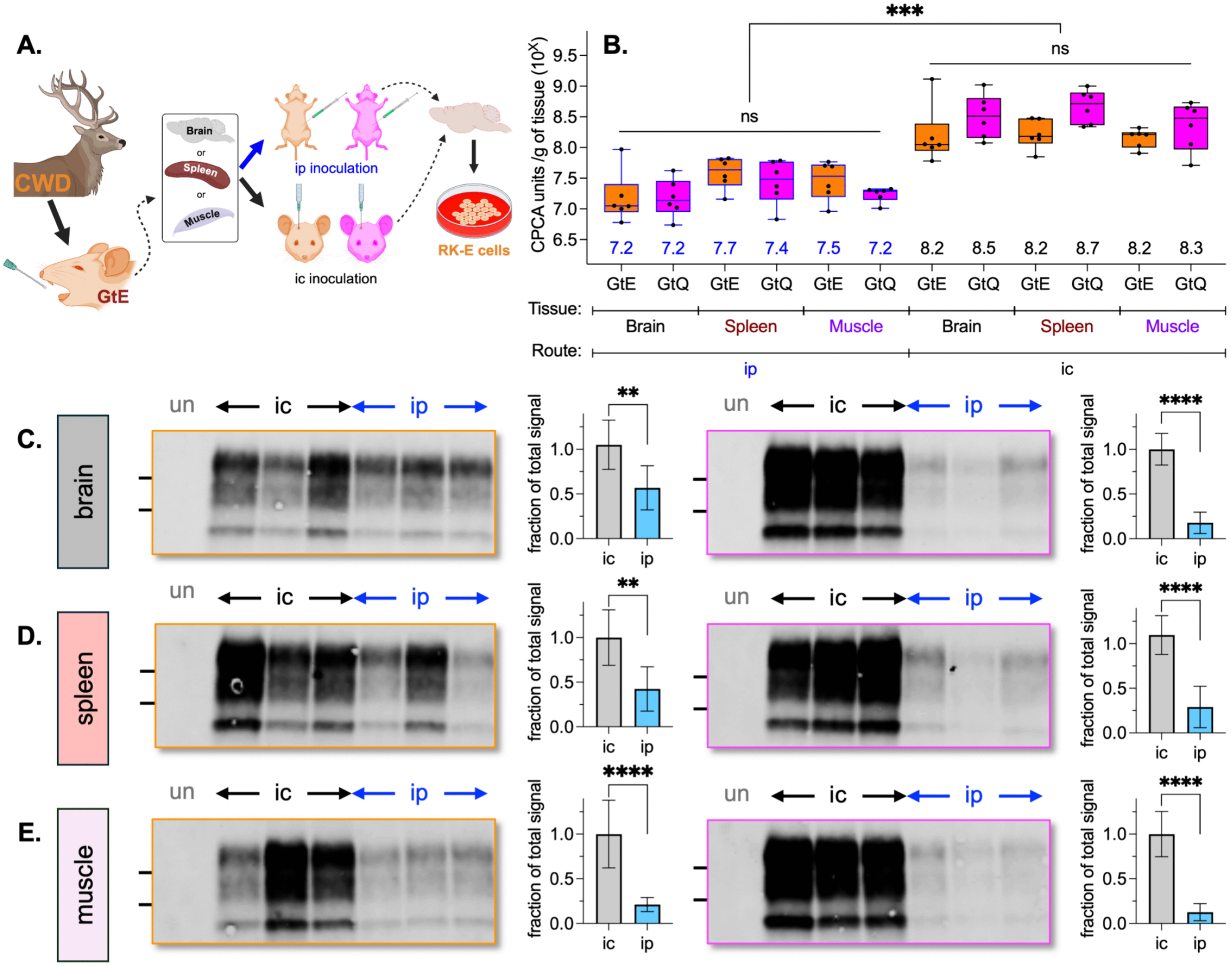
Levels of CWD prions in the brain of Gt mice following intraperitoneal or intracerebral transmissions with diseased brain, spleen or muscle tissue. **A.,** schematic of cervid prion cells assay (CPCA) with infected brain tissue from diseased mice. Created in BioRender. Defranco, **J.** (2026) https://BioRender.com/tomd10m. **B.,** graphical representation of median, minimum, and maximum titers of CWD prions from intraperitoneally (ip, blue) or intracerebrally (ic, black) passaged GtE (orange) and GtQ (magenta) mice. For each condition, three brains were independently analyzed two times, and individual experimental titers are shown as dots. Average titer values for individual brain prions are summarized in S4 Table. Significance is determined by one-way ANOVA (ns, *P* > 0.05; ***, *P* ≤ 0.001). **C. – E.,** representative western blots of PK-treated brain homogenates of diseased GtE and GtQ mice normalized for total protein and quantification of multiple western blots of ip- and ic-inoculated Gt mice. Western blots show CNS tissue of uninfected (un) GtE or GtQ, and Gt mice following ic or ip challenges. The positions of 25 and 20 kDa molecular weight markers are shown. Band signal was computed by densitometric analysis of western blots. Signals from intracerebrally-inoculated mice were averaged and arbitrarily set to be 100% signal. Signals of ip-inoculated mice were averaged and expressed as a fraction of the signal in ic-inoculated mice. Average fractions of total signals are plotted ± SD for 3-5 biological replicates assessed independently two times. Significance was assessed using Student’s unpaired t-test (**, *P* ≤ 0.01 ****; *P* ≤ 0.0001). **C.,** GtE and GtQ mice were challenged with CWD-infected brain homogenate; **D.,** GtE and GtQ mice were challenged with CWD-infected spleen homogenate; **E.,** GtE and GtQ mice were challenged with CWD-infected muscle homogenate. ELISpot plates and western blots were probed with mAb PRC5.

We also monitored the levels of CWD prions in the brains of ip- and ic-inoculated GtE and GtQ mice. We challenged RK13 cells expressing elk PrP (RK-E cells) with brain homogenates from diseased GtE and GtQ mice. Following exposure for 28 days, we used the cervid prion cell assay (CPCA) [47] to assess prion titer (Fig 6A). While brain tissue from ip-inoculated Gt mice harbored prion titers of 7.2 to 7.6 CPCA units/g of homogenate, ic inoculation of GtE and GtQ mice produced elevated titers of 8.2 to 8.7 CPCA units/g of homogenate (Fig 6B and S4 Table). We observed no difference between mice inoculated with prions from either brain, spleen, or muscle tissues. Western blotting of PK-resistant material confirmed that ic-inoculated GtE and GtQ mice had increased levels of CWD prions (Fig 6C-6E). In conclusion, notwithstanding evidence that prions from different tissues possess distinct properties, these analyses demonstrated that the adaptive pressures of the inoculation route were more significant and dictated strain outcomes.

### Convergence of prion conformational properties in cultured cells following infection with CWD prions from brain, spleen, or muscle tissues

**Fig 7:**
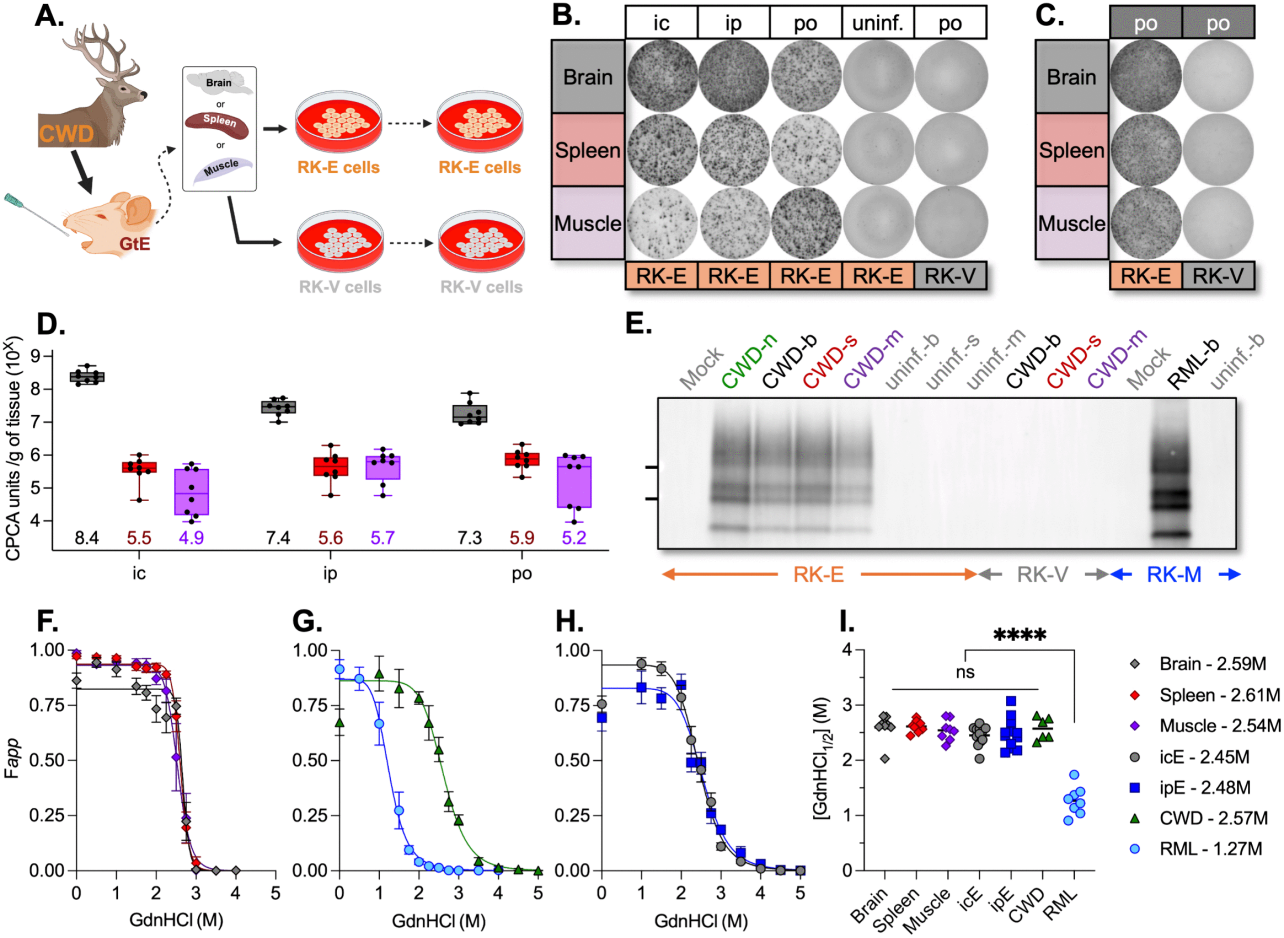
Characterization of prions produced following cell infection of brain, spleen, and muscle homogenates. **A.,** schematic of RK13 cells transfected with elk PrP (RK-E) or an empty vector (RK-V) following infection with brain, spleen, or muscle homogenates from a diseased GtE mouse orally-challenged with elk CWD prions. Dotted arrows indicate the additional passage of RK-E and RK-V cells. Created in BioRender. Defranco, **J.** (2026) https://BioRender.com/tomd10m. **B.** and **C.,** representative wells of an ELISpot plate of RK-E or RK-V cells following prion infection with 0.3% (w/v) of brain, spleen, or muscle tissues from intracerebrally (ic), intraperitoneally (ip), orally (po), or uninfected GtE mice. Cells were plated in **B.,** following 28 days of incubation with GtE homogenates and in **C.,** following five passages. **D.,** graphical representation of median, minimum, and maximum prion titers in RK-E cells following infection of brain, spleen, and muscle GtE tissue derived from ic, ip, or po inoculation. For each condition, three different tissues were independently analyzed two to three times, and individual experimental titers are shown as dots. Average titer values for individual prions are summarized in S5 Table. **E.,** western blotting analyses of PK-treated cell extracts at passage five following prion cell infection with infected tissue, uninfected (uninf.) tissue, or incubation with saline (mock). Tissues infected with CWD prions are derived from brain tissue from a native (n) elk, brain (b) tissue from an orally-challenged GtE mouse, spleen (s) tissue from an orally-challenged GtE mouse, or muscle (m) tissue from an orally-challenged GtE mouse. RK13 cells transfected with mouse PrP which were mock-infected, challenged with brain tissue from a FVB mouse ic-inoculated with RML prions or uninfected brain tissue. The positions of 25 and 20 kDa molecular weight markers are displayed. **F. – I.,** Responses of cell-derived prions to increasing concentrations of GdnHCl. In all cases, Average fractional apparent signal (F*app*) ± SEM after PK digestion is plotted as a function of GdnHCl concentration **(M)**. Sigmoidal dose-response curves were plotted using a four-parameter algorithm. Graphical representations for the concentrations of GdnHCl to produce half-maximal denaturation of prions [GdnHCl_1/2_] are shown in panel **I.** for each condition. **F.,** CWD PrP^Sc^ in RK-E cells originally infected with brain tissue (gray diamonds), spleen tissue (red diamonds), or muscle tissue (purple diamonds). **G.,** CWD PrP^Sc^ in RK-E cells originally infected with native elk brain tissue (green triangles) and RML PrP^Sc^ in RK-M cells originally infected with brain tissue (blue circles). **H.,** CWD PrP^Sc^ in RK-E cells originally infected with brain tissue from ic-inoculated GtE mice (gray circles) or ip-inoculated GtE mice (blue squares). ELISpot plates and western blots were probed with mAb PRC5.

After demonstrating that strain convergence occurs during ip or ic inoculations in Gt mice, we asked whether transmission to other prion systems also results in identical conformational profiles following infection with CWD prions from brain, spleen, and muscle tissues. We challenged RK-E cells with prions from brain, spleen, and muscle tissues from ic-, ip-, and po-inoculated mice (Fig 7A and 7B). In all cases, the total number of living RK-E cells was unaffected by the incubation with infected or uninfected tissues. We observed high levels of PrP^Sc^ following infection of prions from brain, spleen, or muscle tissues, whereas RK-E cells exposed to uninfected homogenates remained devoid of CWD prion accumulation (Fig 7B and 7D). Additionally, the lack of PK-resistant PrP^Sc^ from RK13 cells stably transfected with an empty pIRES-puro3 vector (RK-V cells) following exposure to prions from brain, spleen, and muscle tissues indicated that the positivity in RK-E cells was due to *bona fide* CWD prion replication and not residual inoculum (Fig 7B). While prion titers of brain tissue ranged from 7.0 to 8.6 CPCA units/g of tissue (Fig 7D and S5 Table), brain homogenate from ic-inoculated mice harbored elevated titers compared to its ip- and po-inoculated counterparts, which is in agreement with our previous findings [15] (Fig 6B and S5 Table). Whereas we observed elevated RT-QuIC seeding activity and PMCA amplification efficiency in spleen tissue compared with muscle (Fig 2C, 2D, and 2E), we found no difference in prion titers between spleen and muscle homogenates in RK-E cells (Fig 7D and S5 Table). These tissues also produced a more variable range of prion titers from 4 to 6.1 CPCA units/g of homogenate. We also found no difference in infectivity levels between these two tissues across routes of inoculation, which is consistent with our data using RT-QuIC as an outcome measure (Fig 2C and S2 Table). The demonstration that RK-E cells are susceptible to CWD prions from peripheral tissues of Gt mice is consistent with our findings using CWD prions from retropharyngeal lymph node homogenates of native cervids [44].

We produced chronically infected cells by infecting RK-E cells with 0.3% (w/v) brain, spleen, or muscle homogenates from the same orally infected GtE mouse used for bioassays in Fig 3 (Fig 7A, 7B and S10A Fig). After five passages, we found that RK-E cells harbored high levels of CWD prions, while RK-V cells challenged with the same prion preparations remained devoid of detectable PK-resistant PrP^Sc^ (Fig 7C and 7E). Analyses of the cell lysate by RT-QuIC confirmed these results (S10B Fig). Because brain and peripheral tissues produced distinct prion titers in RK-E cells, we asked if these tissues would produce different levels of infectivity in chronically infected cells. RK-E cells infected with prions from brain, spleen, or muscle tissues produced titers in the range of 10.6 to 11.3 log SD_50_/g of lysate following end-point RT-QuIC dilution analyses (Table 2 and S10C-S10E Fig). We observed no difference in seeding activity between RK-E cells originally infected with different tissues, and levels of infectivity remained comparable for up to nine passages. For comparison, we also generated RK-E cells chronically infected with the original native Elk CWD prions and RK13 cells expressing mouse PrP (RK-M) [48] chronically infected with RML prions (S10A Fig) [15,49]. At passage 5, these chronically infected cells harbored high levels of PrP^Sc^, whereas mock-infected cells remained devoid of prion replication (Fig 7E and S10B Fig). While RK-E cells infected with native CWD prions also produced titers that were comparable to GtE brain, spleen, and muscle infections, we observed ~ 10-fold higher prion titers in RK-M cells infected with RML prions (Table 2 and S10F and S10G Fig). We conclude that RK-E cells harbor comparable prion titers following infection with different sources of CWD prions, while other prion strains can produce distinct titers from identical experimental conditions.

**Table 2:** End-point RT-QuIC titration analyses of cell lysate following infection with prions from brain, spleen, or muscle homogenates at 0.3% (w/v). Each value (log SD_50_ per g of tissue, 10^X^) was computed by Spearman-Kärber analysis. ND, not determined. P. X is the passage number from the initial infection.

	CWD - RK-E cells												RML - RK-M cells		
	po-inoc. muscle			po-inoc. spleen			po-inoc. brain			Native CWD brain			ic-inoc. brain		
	P. 5	P. 7	P. 9	P. 5	P. 7	P. 9	P. 5	P. 7	P. 9	P. 5	P. 7	P. 9	P. 5	P. 7	P. 9
Rep 1	11.4	11.7	11.7	10.4	11.7	11.7	11.0	11.7	10.7	10.7	11.0	11.0	12.7	12.4	13.0
Rep 2	10.7	10.4	11.7	10.4	11.4	10.4	10.7	11.4	11.7	ND	ND	ND	ND	ND	ND
Rep 3	11.7	10.4	10.4	11.0	10.0	11.0	10.7	10.4	11.0	ND	ND	ND	ND	ND	ND
Mean	11.3	10.8	11.3	10.6	11.0	11.0	10.8	11.2	11.1	10.7	11.0	11.0	12.7	12.4	13.0

We assessed the conformational properties of prions from chronically infected cells using the cell-based conformational stability assay (C-CSA) [50]. We observed no differences in the denaturation profiles or [GdnHCl_1/2_] values of RK-E cells infected with brain, spleen, or muscle tissues from po-inoculated GtE mice (Fig 7F and 7I). These indistinguishable conformational properties mirror our CSA experiments using CNS prions, in which the [GdnHCl_1/2_] values were equivalent following transmission of brain, spleen, and muscle tissues following either ip or ic inoculation (Fig 4G). While RK-E cells infected with native elk CWD prions produced an equivalent denaturation profile and [GdnHCl_1/2_] values, prions from RML-infected RK-M cells produced a left-shifted denaturation profile and decreased [GdnHCl_1/2_] value of 1.27 M (Fig 7G and 7I). These analyses of cell-derived prions support our previous comparison of the distinct conformational properties of brain-derived RML and CWD prions [49], and suggest that prions produced in RML-infected RK-M cells and all CWD-infected RK-E cells are composed of different conformers.

We sought additional examples of conformational convergence in RK-E cells following infection with other distinct CWD conformers. We’ve previously demonstrated that CNS prions from ic-inoculated GtE mice (icE) or ip-inoculated GtE mice (ipE) harbor distinct conformational properties, which are heritably propagated upon serial passage to additional GtE mice [15] (S11 Fig). We generated chronically infected RK-E cells by infecting cells with either icE or ipE prions and passaging at least 5 times (S12 Fig). We compared the conformational properties of *bona fide* RK-E CWD prions produced following infection with either icE or ipE and observed indistinguishable denaturation profiles and equivalent [GdnHCl_1/2_] values (Fig 7H and 7I). Collectively, these data indicate that infection of RK-E cells with different CWD prion conformers results in the generation of equivalent prion conformational properties.

## Discussion

Peripheral pathogenesis is a key component of CWD and other prion diseases whose etiology is contagious transmission [51]. In this study, we used Gt mice because they are a refined model for studying prion accumulation in non-CNS tissues and peripheral pathogenesis. Some previous Tg models allowed assessment of LRS prion accumulation [23]; however, random transgene integration in these mice results in disparate levels of PrP and variable expression across different cell types [52]. Prion protein overexpression may obscure interpretation of these findings, as studies have shown that this feature of Tg mice can alter prion strain phenotypes [53,54]. The use of a gene-targeted approach results in endogenous expression of foreign PrP throughout the organism [11–13] and allows for authentic interactions between prions and PrP^C^ in their native cellular states. We observed that the localization of CWD prions in the spleen and skeletal muscles of diseased GtE and GtQ mice is reminiscent of previous findings of lab-adapted prion strains in wild-type mice [31–34,37,55]. Whereas prion strains can have distinct PrP^Sc^ deposition profiles in the CNS, the similar deposition patterns in peripheral tissues suggests a common mechanism of prion accumulation. Further investigation of non-CNS tissues in additional Gt mice infected with other prion diseases, such as scrapie and sCJD [53,56], could augment our understanding of peripheral prion replication.

Our previous studies showed that following transmission of CWD prions from native cervids, the route of inoculation alters the conformational and biochemical properties of prions in the brain, which leads to different neuropathological phenotypes and levels of prion replication in the CNS [15]. Additionally, we observed accelerated incubation periods upon serial passage of only ic inoculated mice, indicating that the properties of native CWD are modified following direct prion injection into the brain. These data suggest that different tissues contain cofactors that select for distinct prion strains. Building on these findings, this study aimed to investigate the strain properties of CWD prions in different tissues. We found that the biochemical and conformational properties of the prions within the brain and peripheral tissues were distinct. Despite replication using the same host PrP^C^ sequence, these findings demonstrate that prions residing in different cells or tissues can exhibit distinct properties. Our results support previous evidence that prions are composed of clouds of distinct conformers – or conformational “islands” – that can vary in prominence across different cell types [57–60]. Several studies have shown that specific purified cofactors within PMCA reactions can produce different prion strains with distinct biochemical and glycoform profiles [61–64]. Our findings that muscle homogenate devoid of PrP can hinder PMCA amplification are consistent with these observations and suggest that tissue-specific cofactors can exert unique pressures on prion replication. Additional support for this idea comes from our finding that indistinguishable prion conformers are produced following infection of RK-E cells with conformationally distinct ip- or ic-passaged CWD prions, suggesting that this is a result of a specific microenvironment within RK13 cells. These data mirror the demonstration that the tissue origin can influence the sialylation status of PrP^Sc^ [65]. Additionally, we found that the endogenous proteolytic processes of PrP^C^ in the spleen were distinct from those in the brain and muscle. Because these endoproteolytic events and glycosylation of PrP^C^ were seemingly unrelated to the different prion glycoforms, future research should focus on understanding the mechanisms that control the endoproteolytic processes of PrP^C^ and their relationship to the prions that replicate in different tissues. Collectively, these findings suggest that different cell types within the same infected host can affect the replicative, biochemical, and conformational properties of prions.

Transmissions of prions composed of distinct conformers, such as ip- and ic-inoculated CWD prions, retain their conformational differences upon corresponding passage to the same host [15]. These data bolster a large body of evidence that demonstrates that the conformational properties of PrP^Sc^ encipher prion strains [20,22,66–68]. Consequently, we expected that the distinct prion conformers that we detected in the brain and peripheral tissues would produce different strain properties upon transmission to additional Gt mice. Thus, it was unexpected to observe indistinguishable transmission profiles and strain properties following challenges of CWD prions from brain, spleen, and muscle homogenates. Moreover, compared with peripheral tissues, diseased brain tissue harbors higher levels of PK-resistant PrP^Sc^, greater *in vitro* amplification efficiency by PMCA, increased RT-QuIC seeding activity, and higher infectious titers upon infection of RK-E cells. Despite these differences, brain, spleen, and muscle homogenates produced comparable incubation times in GtE and GtQ mice. Further investigations, for example using endpoint titrations of CWD prions from brain and peripheral tissues, may provide insights into the basis of this discrepancy. We also observed identical conformational properties of prions produced in RK-E cells following infection with CWD prions from brain or peripheral tissues. Collectively, our findings are innovative, as they suggest that although distinct conformers may be present in different tissues within infected animals, transmission of these tissues results in identical disease outcomes and prion strain properties. Because we still observed distinct disease outcomes following ip or ic transmission of spleen and muscle homogenates, the data in this study also support our previous findings that the route of inoculation influences strain outcome in CWD-infected GtE and GtQ mice [15]. Because we also demonstrated that similar levels of seeding activity and infectivity of prions are present in spleen and muscle tissue following both peripheral and ic challenges, these data suggest that prion replication in the spleen and muscle is unaffected by the route of inoculation. We therefore speculate that these “islands” of prion conformers emerge following distinct pressures within different host compartments.

The majority of CWD prion strain analyses have been conducted with diseased brain tissue [11,15,38,69–73]. This may not reflect what is occurring in nature, as natural transmission of CWD between North American cervid species or other mammals is most likely caused by prions shed from peripheral tissues, the lymphatic system, or bodily fluids. This study demonstrates that Gt mice can be used to assess the properties of mammalian prions in peripheral tissues and assess their transmission profiles to additional Gt mice and susceptible cell culture systems. Because the study reported here was successful in understanding the properties of North American CWD prions, it warrants further investigation of other prion strains in peripheral tissues. Because of the diversity and instability of CWD strains in Northern Europe [45,74,75], determining the replicative capacity of these prions in the LRS, skeletal muscle, and other peripheral tissues could inform the risk of handling or consuming tissues from diseased cervids. Furthermore, whether these “islands” of prion conformers across various tissues yield divergent outcomes following interspecies transmission requires investigation in additional Gt mice encoding for human and other mammalian prion proteins [76]. Because the zoonotic potential of CWD has primarily been evaluated following ic transmission of brain tissue, this study provides proof of concept to model natural transmission scenarios. For example, feeding Gt mice expressing human PrP with muscle from native CWD prions, or CWD prions that have adapted following transmission in GtE and GtQ mice [45], is of high priority.

## Materials and methods

### Ethics statement

All animal work was performed in an AAALAC-accredited facility in compliance with the Guide for the Care and Use of Laboratory Animals. All procedures used in this study were performed in compliance with the Colorado State University Institutional Animal Care and Use Committee. Breeding number, 5405; Study number, 4710.

### CWD isolates

Frozen brains of diseased elk, referred to as 99W12389, and deer, referred to as D10 from the United States [11,38] were homogenized in phosphate buffered saline (PBS) lacking calcium and magnesium ions at 10% (w/v).

### Animal models and disease transmission

The generation and characterization of gene-targeted mice expressing elk PrP or deer PrP, referred to as GtE and GtQ mice, respectively, have been described previously [11]. During primary passage of CWD isolates, mice were challenged by intracerebral or intraperitoneal routes of inoculation or *per os* (po) by oral gavage. Six to eight-week-old mice were anesthetized with isoflurane prior to free-hand inoculations. For intracerebral inoculations, 30 µl of 1% brain homogenate was injected into the right parietal cortex. For intraperitoneal inoculations mice were injected with 100 µl of 1% brain homogenate. For oral gavage, mice were challenged with 100 µl of 1% brain homogenate using a 20 g 1.5 inch feeding needle. Twelve serial transmissions were conducted using infected brain, spleen, or muscle homogenates diluted to 1% (w/v). Four transmissions for each brain, spleen, and muscle tissues were conducted using GtE or GtQ mice by either the intracerebral or intraperitoneal routes as described above. Animals were maintained on an alternating 12-hour (h) light and 12-h dark cycle with free access to food and water. The time to disease onset, or incubation time, is the time between the date of inoculation and the first date on which definitive, subsequently progressive clinical signs were identified. Mice dying from intercurrent illnesses at earlier stages were excluded from calculations. Inoculated mice were assessed twice weekly for the onset of neurological signs by two different investigators [15]. Videos of prion sick mice were also taken and reviewed by two investigators to confirm clinical signs. For clarity, truncal ataxia refers to any clinical sign encompassing loss of coordination, loss of grip strength, impaired balance while the mouse is stationary or walking, and other gait abnormalities that are not related to balance such as flattened gait. Impaired balance is assessed while the mouse is stationary or walking. Flattened gait is characterized by a depressed and lowered posture of the mouse while walking. Modified gait is identified by any condition that results in the limitation – usually in the hindlimbs – or inability of the mouse to walk. Mice were humanely killed at the terminal stage of disease. The muscles of a subset of diseased mice were removed and frozen for biochemical analyses. The muscles of additional diseased mice were removed and immersion fixed in formalin for histopathological assessments. The spleens of a subset of diseased mice were removed and frozen for biochemical analyses. The spleens of additional diseased mice were removed and immersion fixed in formalin for histopathological assessments. The brains of a subset of diseased mice were removed and bisected down the midline, one hemisphere being frozen for biochemical analyses, and the other immersion fixed in formalin for neuropathological assessments. The whole brains of additional diseased mice were removed and either immersion fixed in formalin for more complete neuropathological assessments. We processed coronal sections for all formalin-fixed brain tissues.

### Tissue preparation for PrP^Sc^ analyses

Total protein content was assessed by the bicinchoninic acid (BCA) assay (ThermoFisher Scientific) for all tissues and cell lysate. For Fig 1F, all tissues were prepared under identical conditions prior to SDS-PAGE. Homogenates were treated with 1 mg/ml DNase and 5mM MgCl_2_ for 30 min at 37 °C prior to PK digestion with 50 µg/ml PK and a final volume of 4% sarkosyl for 1 h at 37 °C. Digestion was terminated with 2 mM phenylmethylsulfonyl fluoride (PMSF) for 10 min at room temperature and subjected to 100,000 x g spin for 1 h at 4 °C. The supernatant was discarded and the resulting pellet was denatured in SDS-PAGE loading buffer at 100 °C for 10 min. For tissues in the western blot in Figs 1K and S6, brain, spleen, and muscle homogenates were treated with Pronase E (final concentration of 100 µg/ml) and incubated for 30 min at 37 °C. Digestion was terminated with EDTA (final concentration of 10 mM). Prior to precipitation, samples were treated with 50 U/mL Benzonase and 2% sarkosyl for 10 min at 37 °C. Samples were treated with sodium phosphotungstic acid (NaPTA) for a final concentration of 0.3% (w/v) for 30 min at 37 °C. Following incubation, iodixanol and NaPTA were added to produce a final concentration of 35% (w/v) and 0.3% (w/v), respectively, and subjected to centrifugation for 90 min at 16,100 x g at 4 °C. 520 µL of the supernatant was loaded into Ultrafree-HV microcentrifuge filtration unit (0.45 μm pore size Durapore membrane, Millipore, Prod. No. UFC30HV00) and subjected to centrifugation for 30 sec at 12,100 x g. 480 µL of filtered samples were treated with sarkosyl (final concentration of 1% [w/v]) and NaPTA (final concentration of 0.15% [w/v]) for 10 min at 37 °C. Samples were subjected to centrifugation for 90 min at 16,100 x g at 4 °C. The remaining pellet was resuspended in 0.1% (w/v) sarkosyl in PBS. SDS-PAGE loading buffer was added to the samples and denatured at 100 °C for 10 min. For PMCA products in Figs 2D, 2G and S7, sonicated samples were treated with 100 µg/ml PK in the presence of 2% sarkosyl for 1 h at 60 °C. Digestion was terminated with SDS-PAGE loading buffer, and samples were denatured at 100 °C for 10 min. For PK-treated samples in Figs 6C-6E and S6, brain homogenates or sample preparations were treated with 50 µg/ml PK in the presence of 2% sarkosyl for 1 h at 37 °C. Digestion was terminated with SDS-PAGE loading buffer, and samples were denatured at 100 °C for 10 min. For quantification in Fig 6D-6E, signals from ic-inoculated Gt mice were averaged and arbitrarily set as 100%, and signals of ip-inoculated Gt mice were averaged and expressed as a fraction of the ic-inoculated signal. Uninfected Gt homogenate lanes were set to 0%. For western blots in Figs 7E and S12B, cultured cell monolayers were harvested using mechanical dissociation with cold lysis buffer (50 mM Tris-HCl, pH 8.0, 150 mM NaCl, 0.5% sodium deoxycholate, 0.5% Igepal CA-630). Cell lysates were treated with 20 µg/ml PK for 1 h at 37 °C. Digestion was terminated with 2 mM PMSF for 10 min at room temperature and subjected to 100,000 x g spin for 1 h at 4 °C. The supernatant was discarded and the resulting pellet was denatured in SDS-PAGE loading buffer at 100 °C for 10 min.

### De-glycosylation of PrP^C^

Proteins were de-glycosylated with PNGase F as specified by the supplier (New England Biolabs). All tissue homogenates in the western blots in Fig 1B and 1C were incubated at 95 °C for 10 minutes with 1 µL of 10X glycoprotein denaturing buffer for a total volume of 10 µL. Following cooling the samples on ice for five minutes, a mixture of 2 of µL of 10% NP-40, 2 µL of 10X Glycobuffer, and 5 µL of H2O were added to all samples. For the PNGase F treated samples (+), 1 µL PNGase F was added, while 1 µL of H2O were added for untreated samples (-). Enzymatic reactions were terminated by adding SDS-PAGE loading buffer.

### Western blotting

Denatured tissue and cell lysate samples were subjected to SDS-PAGE using precast 12% discontinuous Bis-Tris gels (Bio-Rad Laboratories). Proteins were transferred to Immobilon-FL PVDF membranes (EMD Millipore) and blocked with 5% non-fat milk for 1–2 h. Blots were treated with mAb PRC5 [77] (1:5000) or mAb SAF32 (Caymen Chemical) (1:1000) followed by IRDye 800CW Goat anti-Mouse IgG (H + L) (LI-COR Biosciences, Lincoln, NE, USA) (1:25,000) for 1 h. Membranes were washed with tris-buffer saline (TBS) and tris-buffer saline/tween (TBST) and scanned using the LICOR Odyssey DLx imaging system (LI-COR Biosciences, Lincoln, NE, USA). Signals were analyzed using LI-COR Image Studio Acquisition Software.

### Immunohistochemical (IHC) analyses

Brains, spleens, and muscles were embedded in paraffin blocks and 5 µm sections were placed onto positively charged glass slides. Slides were heated to 60 °C for 30 min. Slides were treated with xylene and graduated ethanol treatment followed by treatment with 88% formic acid for 30 min. Antigen retrieval was conducted using citrate buffer in the 2100 Retriever (Proteo-Genix, Schiltigheim, France). Slides were blocked in 5% non-fat milk for 30 min at room temperature. For anti-PrP staining, slides were probed with Fab D18 diluted at 1∶2500 in PBST overnight at 4 °C, followed by exposure to biotin labelled goat Fab anti-human IgG secondary antibody (Southern Biotech, Birmingham, AL) at a 1:2500 dilution for 1 h at room temperature. The D18 antibody was produced in-house from cultured Chinese hamster ovary (CHO) cells provided by Richard Bessen (Colorado State University, Colorado, USA) [78]. For cell-type specific staining in the spleen, slides were probed with Purified Rat Anti-Mouse Follicular Dendritic Cell (Clone FDC-M1, BD Biosciences) (1:50) or Purified Rat Anti-Mouse CD21/CD35 (Clone 7G6, BD Biosciences) (1:1000), followed by use of the Anti-Rat Ig HRP Detection Kit per the supplier’s instructions (BD Biosciences). Staining was developed using avidin-conjugated horseradish peroxidase (HRP) with diaminobenzidine (DAB) as substrate (Vector Laboratories, Burlingame, CA) for 30 minutes.

### Real-time quaking-induced conversion (RT-QuIC)

The expression plasmid containing the truncated Syrian hamster PrP^C^ coding sequence (90–231) was provided by Dr. Byron Caughey (Laboratory of Persistent Viral Diseases, Rocky Mountain Laboratories, Hamilton, MT). Recombinant protein purification and RT-QuIC analyses were performed as previously described [46]. RT-QuIC end point titration analyses (log SD_50_/g of brain tissue) were calculated using the Spearman-Kärber method as previously described [41]. Tissue homogenates and cell lysate were diluted in 0.1% SDS/PBS.

### Protein misfolding cyclic amplification (PMCA)

Infected GtE tissue homogenates were used to seed PMCA reactions. Brains from uninfected, perfused GtE mice and brains and muscles from uninfected, perfused *Prnp*^*-/-*^ mice [42,43] were homogenized in PMCA conversion buffer (1X phosphate-buffer saline, 150 mM sodium chloride, 1% Triton X-100, protease inhibitor tablet (cOmplete Protease Inhibitor Mini, Roche), 12 mM EDTA, 0.05% digitonin]. Infected brain, spleen, and muscle homogenates were added to PMCA substrates. Samples were sonicated using the QSonica700 sonicator at ~ 120 watts/second and subjected to one round of PMCA (288 sonication/incubation cycles; 10 seconds of sonication, 14 min, and 50 seconds rest). For Fig 2G-2I, 10 µL of perfused brain or muscle homogenates from *Prnp*^-/-^ mice were mixed with different ratios of GtE perfused brain homogenates and infected seeds (depicted in S8 Fig).

### Conformational Stability Assay (CSA)

For Fig 1J, insoluble PrP was extracted from brain and spleen homogenates prior to treatment with guanidine hydrochloride (GdnHCl). Brain homogenate from one animal was used for each replicate and spleen homogenates from two animals were pooled for each replicate. Following a brief spin (5,000 x g spin for 5 min at 4 °C) to pellet the debris, brain and spleen homogenates were treated with 1 mg/ml DNase and 5mM MgCl_2_ for 1h at 37°C with gentle agitation. Samples were treated with sarkosyl (final concentration of 2% [w/v]) for 30 min at 37°C with gentle agitation. Following a brief spin (5,000 x g spin for 5 min at 4 °C), the supernatants were subjected to 100,000 x g spin for 1 h at 4 °C. The pellets of either brain or spleen were resuspended in 0.1% (w/v) sarkosyl in PBS and pooled together prior to treatment with GdnHCl. The insoluble pellets were incubated for 30 min at 37 °C to disassemble aggregates. Samples were treated with GdnHCl at a ratio of 1:3 for 1 h at room temperature with gentle agitation. Samples were adjusted with PBS to 0.4 M GdnHCl and treated with PK (final concentration of 50 µg/ml) and sarkosyl (final concentration of 2% [w/v]) for 1 h at 37 °C. Digestion was terminated with 2 mM PMSF for 10 min at room temperature and subjected to 100,000 x g spin for 1 h at 4 °C. The supernatant was discarded and the resulting pellet was denatured in SDS-PAGE loading buffer at 100 °C for 10 min. Samples were subjected to western blotting analyses as described above. All other CSA analyses on brain tissues were conducted as previously described [40]. To normalize for PrP^Sc^ signal, brains with higher levels (determined by western blotting) were diluted in FVB *Prnp* knockout mouse brains. Samples containing 15 µg protein were incubated with increasing concentrations of GdnHCl in 96-well plates for 1 h at room temperature. Samples were adjusted with PBS to 0.4 M GdnHCl and transferred onto nitrocellulose membranes (Whatman GmbH, Dassel, Germany) using a dot blot apparatus. After two PBS washes, membranes were air-dried for 1 h, then incubated with 5 µg/ml PK in cell lysis buffer for 1 h at 37 °C. PK was inactivated with 2 mM PMSF. Membranes were treated with 3 M guanidine thiocyanate (GTC)/20mM Tris-HCl, pH 7.8 for 10 min at room temperature. After four washes with PBS, membranes were blocked with 5% Bio-rad Blotting-Grade Blocker in TBST for 1 h, probed with mAb PRC5 [77] diluted in TBST 1∶5000 overnight at 4 °C, followed by IRDye 800CW Goat anti-Mouse IgG (H + L) (LI-COR Biosciences, Lincoln, NE, USA) (1:25,000) for 1 h. Membranes were washed with TBST and TBS and scanned using the LICOR Odyssey DLx imaging system (LI-COR Biosciences, Lincoln, NE, USA). Signals were analyzed using LI-COR Image Studio Acquisition Software.

### Lesion profiling

Assessments were conducted as previously described [79]. Coronal sections of paraffin-embedded mouse brains were stained with hematoxylin and eosin. Two investigators assessed the severity of vacuolar degeneration in cerebral gray matter from nine regions by light microscopy. The scale of vacuolation severity was assessed in each region with a zero score reflecting no pathology and five representing maximum pathology.

### Cervid prion cell assay

RK13 cells engineered to stably express elk PrP (RK-E), mouse PrP (RK-M), and transfected with an empty vector (RK-V) were maintained in Dulbecco’s Modified Eagle Medium containing 10% (w/v) fetal bovine serum and 1 μg/ml penicillin/100 U/ml streptomycin. CWD titers were assessed after infection of RK-E cells with dilutions of tissue homogenate. For Figs 6A, 6B and S12 and S4 Table, crude homogenates were diluted in PBS. For Fig 7A and 7B and S5 Table, tissue homogenates were diluted in PBS following a brief spin (5,000 x g spin for 5 min at 4 °C) to pellet cell debris. Following incubation with tissue homogenates for 28 d, 20,000 cells were transferred to ELISpot plates (PVDF/EMD Millipore). Wells were incubated with 5 µg/ml PK in cell lysis buffer for 1.5 h at 37 °C. PK was inactivated with 2 mM PMSF. Membranes were denatured in 3 M GTC/10mM Tris-HCl, pH 8.0 for 10 min at room temperature. After four washes with PBS, membranes were blocked with SuperBlock Blocking Buffer (ThermoFisher Scientific) for 1 h. ELISpot plates were probed with mAb PRC5 (1∶5000) overnight at 4 °C, followed by alkaline phosphatase conjugated goat anti-mouse IgG (Southern Biotech). Immunoreactivity was visualized using 5-bromo-4-chloro-3-indolylphosphate (BCIP)/ nitro blue tetrazolium (NBT) substrate (Roche). Spots were imaged and quantified with the CTL Immunospot Imager. Chronically infected cells were maintained in identical conditions as naïve cells. For C-CSA analyses, prior to treating wells with PK, increasing concentrations of GdnHCl were added to wells for 1 h at room temperature. Fractional apparent signal (*Fapp)* was calculated by the computing the total number of spots for each GdnHCl concentration.

*Statistical analyses* were conducted using GraphPad Prism software (San Diego, CA).

## Supporting information

S1 Fig PrP^C^ expression in brain, spleen, and muscle tissue of GtE mice. Western blotting of tissue homogenates from uninfected GtE mice. The amount of protein loaded (µg) for each tissue homogenates is shown. The positions of 37, 25, 20, and 15 kDa molecular weight markers are shown and blot was probed with mAb PRC5.
(TIF)

S2 Fig A., Examples of locations of prion glycoform species used for quantification from Fig 1F. Quantifications are plotted in Fig 1E. **B.,** the western blot shown in Fig 1F at different levels of exposure. The positions of 25 and 20 kDa molecular weight markers are shown and the blot was probed with mAb PRC5.
(TIF)

S3 Fig Immunohistochemical analyses of PrP in splenic tissue of Gt and *Prnp^-/-^* mice. Formalin-fixed tissues were probed with anti-PrP Fab d18. Tissues were collected from uninfected GtE mice, or *Prnp*^*-/-*^ mice, or GtE and GtQ mice intracerebrally or intraperitoneally-inoculated with CWD prions. All images were taken from the germinal centers of the spleen. Scale bars, 20 µM.
(TIF)

S4 Fig Immunohistochemical analyses of splenic tissue from an ip-inoculated GtE mouse. Serial sections of the same formalin-fixed splenic tissue were probed with **A., B.,** anti-follicular dendritic cell (FDC) Ab FDC-M1, **C., D.,** anti-CD21/CD35 Ab 7G6 or **E.,** hematoxylin and eosin. Panels **A.** and **C.** are complete, uncropped versions of images in Fig 1H. Thin scale bars, 100 µM; bold scale bars, 20 µM. A key for the numbered anatomical regions is shown.
(TIF)

S5 Fig Immunohistochemical analyses of muscle tissue from CWD-infected and uninfected mice. **A.,** high magnification images of muscle fibers and muscle spindles from uninfected *Prnp*^*-/-*^, uninfected GtE mice, ic-inoculated GtE mice, or ip-inoculated GtE mice. The images from the ip-inoculated mouse are from the same mouse as Fig 1J. Scale bars, 10 µM. **B.,** lower magnification images of the same formalin-fixed muscle tissue from the ic-inoculated mouse in panel A. Anatomical regions are shown: S, muscle spindle; F, muscle fiber. Muscle spindles were identified by the typical fibrous capsule which surrounds nuclear bags. Scale bars, 50 µM. Tissues were probed with anti-PrP Fab d18. In some cases, serial sections following hematoxylin and eosin staining are also shown.
(TIF)

S6 Fig Comparison of crude and NaPTA-precipitated brain homogenate preparations. Western blotting of tissue homogenates from uninfected (un) or intraperitoneally (ip)-inoculated GtE mice. Crude brain homogenates and preparations following NaPTA precipitation were either untreated or treated with proteinase-K (PK). The positions of 37, 25, 20, and 15 kDa molecular weight markers are shown and blot was probed with mAb PRC5.
(TIF)

S7 Fig Protein misfolding cyclic amplification (PMCA) reactions seeded with uninfected tissues. Representative western blots showing the PMCA products following proteinase K (PK) treatment as indicated. Reactions were seeded with uninfected brain, spleen, or muscle tissue as indicated. Non-amplified brain homogenate controls are shown: GtE, uninfected GtE brain homogenate; CWD, elk CWD prion-infected GtE mouse brain homogenate. PMCA products of serial dilutions 10^-2^ to 10^-15^ % (w/v) of CWD seed are shown in bold. The positions of 25 and 20 kDa molecular weight markers are shown and all western blots were probed with mAb PRC5.
(TIF)

S8 Fig Schematic for experimental design for Fig 2G and 2H. Perfused GtE brain homogenate (yellow) was either seeded with CWD-infected muscle from GtE mice (purple) or CWD-infected brain from GtE mice (dark gray). Seeds were diluted by 10-fold in each reaction. To assess the conversion ability in the presence of different tissues, 10% (w/v) of perfused muscle (red) or brain (light gray) from *Prnp*^*-/-*^ (KO) mice was added to the reaction. Created in BioRender. Defranco, J. (2026) https://BioRender.com/tomd10m.
(TIF)

S9 Fig Responses of native CWD prions to increasing concentrations of GdnHCl. Elk CWD (E-CWD) prions are shown in orange and deer CWD prions (Q-CWD) are shown in magenta. One biological replicate was assessed independently three times. Average fractional apparent signal (F*app*) ± SEM after PK digestion is plotted as a function of GdnHCl concentration (M). Sigmoidal dose-response curves were plotted using a four-parameter algorithm. The concentrations of GdnHCl to produce half-maximal denaturation of prions [GdnHCl_1/2_] are shown.
(TIF)

S10 Fig Amyloid seeding of prions produced from chronically-infected RK-13 cells following transmission of different prion strains. **A.,** schematic of generation of chronically-infected RK-E and RK-M cells following transmissions of CWD or RML prions, respectively. Brain, spleen, and muscle graphics depict CWD-infected tissues from an orally-inoculated GtE mouse. Created in BioRender. Defranco, J. (2026) https://BioRender.com/tomd10m. **B.,** assessment of amyloid seeding of chronically infected RK-13 cells after five passages following incubation with inocula for 28 days. Filled circles represent cells initially incubated with prion-positive homogenates. Open circles represent cells initially incubated with prion-negative homogenates. The source of the homogenate is shown. Direct samples (filled and dotted symbols) represent RT-QuIC reactions with CWD-infected tissues. **C - G.** endpoint dilution RT-QuIC analyses of chronically infected RK-13 cells at various passages expressed as P.5, P.7, etc. Titration analyses are shown in Table 2. **C.**, RK-E cells originally infected with CWD-infected brain homogenate from an orally inoculated GtE mouse. **D.,** RK-E cells originally infected with CWD-infected spleen homogenate from an orally inoculated GtE mouse. **E.,** RK-E cells originally infected with CWD-infected muscle homogenate from an orally inoculated GtE mouse. **F.,** RK-E cells originally infected with naive elk CWD brain homogenate. **G.,** RK-M cells originally infected with RML-infected brain homogenate from an ic-inoculated FVB mouse. For **B. - G.,** data is expressed as the Rate of Amyloid Formation, which is the inverse of the time to threshold.
(TIF)

S11 Fig Conformational stabilities of native elk CWD prions and its derivatives following peripheral or intracerebral challenges. New analyses of data from (15). Graphical representation of the concentrations of GdnHCl to produce half-maximal denaturation of prions [GdnHCl_1/2_] are shown. Passage numbers are expressed as p1, p2, etc., throughout. Significance is determined by one-way ANOVA (ns, *P* > 0.05; ****, *P* ≤ 0.0001).
(TIF)

S12 Fig Generation of chronically infected cells following transmission of native elk CWD and its derivatives following intraperitoneal or intracerebral challenges. **A.,** schematic of generation of chronically infected RK-E cells. Brain homogenate from a North American elk CWD isolate, intracerebrally-inoculated GtE mouse (icE), or intraperitoneally-inoculated GtE mouse (ipE) incubated with RK-E cells for 28 days. Cells were harvested and repeatedly passaged. Created in BioRender. Defranco, J. (2026) https://BioRender.com/tomd10m. **B.,** western blot showing chronically-infected RK-E cells at passage 10. Samples were either untreated (20 μg of protein) or treated (1 mg of protein) with PK as indicate and immunoblots were probed with mAb PRC5. The positions of 37, 25, and 20 kDa molecular weight markers are shown.
(TIF)

S1 Table Transmission of North America Elk CWD prions (99W12389) to GtE mice following intracerebral or peripheral routes of inoculation. Time to disease onset (incubation time) is expressed as the mean time, in days, at which inoculated mice first developed ultimately progressive signs of neurological disease. Variance is expressed as ± standard error of the mean (SEM). Prion disease was confirmed by western immunoblotting, histoblotting, or immunohistochemical analyses of CNS prions. Mice dying of intercurrent illnesses prior to prion disease onset were excluded from these calculations. Transmissions previously reported in [11,13].
(DOCX)

S2 Table RT-QuIC endpoint titration analyses of brain, spleen, and muscle of diseased-GtE mice following intracerebral (ic), intraperitoneal (ip), or oral gavage (po) routes of inoculation. In each case, three biological replicates (Rep.) were assessed, and the mean was computed. Each value (log SD_50_ per g of tissue, 10^X^) was computed by Spearman-Kärber analysis.
(DOCX)

S3 Table Three-way ANOVA analyses of times to disease onset of GtE or GtQ mice following intracerebral (ic) or intraperitoneal (ip) inoculation with either brain, spleen, or muscle tissue. SS, sum-of-squares; DF, degrees of freedom; MS, mean squares; F, F ratio; DFn, degrees of freedom numerator; DFd, degrees of freedom denominator.
(DOCX)

S4 Table CPCA titration analyses of brain tissue of GtE and GtQ mice following intracerebral or intraperitoneal inoculation with infected brain, spleen, or muscle tissue (CPCA units per g of tissue, 10^X^). (DOCX)

S5 Table CPCA titration analyses of brain, spleen, and muscle of diseased-GtE mice following intracerebral (ic), intraperitoneal (ip), or oral gavage (po) routes of inoculation (CPCA units per g of tissue, 10^X^). (DOCX)

S6 Table Transmission of CWD-infected brain, spleen, and muscle homogenate to GtE and GtQ mice. Time to disease onset (incubation time) of individual mice, in days, at which they first developed ultimately progressive signs of neurological disease. Mice dying of intercurrent illnesses prior to prion disease onset were excluded.
(XLSX)
