# Supplementary material for: Distinct prion conformers from brain and peripheral tissues of gene-targeted mice produce convergent CWD strain properties
Source: PLoS Pathog. 2026 Jun 4;22(6):e1014303. doi: 10.1371/journal.ppat.1014303 (PMC13252839; doi:10.1371/journal.ppat.1014303)
Supplement: S1 Table — Time to disease onset (incubation time) is expressed as the mean time, in days, at which inoculated mice first developed ultimately progressive signs of neurological disease. Variance is expressed as ± standard error of the mean (SEM). Prion disease was confirmed by western immunoblotting, histoblotting, or immunohistochemical analyses of CNS prions. Mice dying of intercurrent illnesses prior to prion disease onset were excluded from these calculations. Transmissions previously reported in [11,13]. (DOCX) [file ppat.1014303.s013.docx]

| **Transmission Route** | **Primary passage** |
| --- | --- |
| Intraperitoneal (ip) | 313±10 (13/13) |
| Oral Gavage (po) | 307±3 (13/13) |
| Intracerebral (ic) | 213±6 (14/14) |
