# Supplementary material for: Distinct prion conformers from brain and peripheral tissues of gene-targeted mice produce convergent CWD strain properties
Source: PLoS Pathog. 2026 Jun 4;22(6):e1014303. doi: 10.1371/journal.ppat.1014303 (PMC13252839; doi:10.1371/journal.ppat.1014303)
Supplement: S2 Table — In each case, three biological replicates (Rep.) were assessed, and the mean was computed. Each value (log SD50 per g of tissue, 10X) was computed by Spearman-Kärber analysis. (DOCX) [file ppat.1014303.s014.docx]

| **Route** | **Brain** | | | | **Spleen** | | | | **Muscle** | | | |
| --- | --- | --- | --- | --- | --- | --- | --- | --- | --- | --- | --- | --- |
|  | Rep. 1 | Rep. 2 | Rep. 3 | **Mean** | Rep. 1 | Rep. 2 | Rep. 3 | **Mean** | Rep. 1 | Rep. 2 | Rep. 3 | **Mean** |
| **ic** | 11.8 | 12.0 | 12.3 | **12.0** | 10.3 | 9.8 | 10.0 | **10.0** | 7.0 | 7.8 | 7.3 | **7.3** |
| **ip** | 11.0 | 11.3 | 11.0 | **11.1** | 10.3 | 10.0 | 10.3 | **10.2** | 7.8 | 8.0 | 7.7 | **7.8** |
| **po** | 10.8 | 11.0 | 11.3 | **11.0** | 9.3 | 10.3 | 10.0 | **9.9** | 8.3 | 7.8 | 8.3 | **8.1** |
