# Supplementary material for: Distinct prion conformers from brain and peripheral tissues of gene-targeted mice produce convergent CWD strain properties
Source: PLoS Pathog. 2026 Jun 4;22(6):e1014303. doi: 10.1371/journal.ppat.1014303 (PMC13252839; doi:10.1371/journal.ppat.1014303)
Supplement: S3 Table — SS, sum-of-squares; DF, degrees of freedom; MS, mean squares; F, F ratio; DFn, degrees of freedom numerator; DFd, degrees of freedom denominator. (DOCX) [file ppat.1014303.s015.docx]

| **Source of Variation** | **% of total variation** | **P value** |
| --- | --- | --- |
| tissue | 5.833 | <0.0001 |
| (ic vs ip) | 4.904 | <0.0001 |
| (GtE vs GtQ) | 65.36 | <0.0001 |
| tissue x (ic vs ip) | 2.113 | 0.0066 |
| tissue x (GtE vs GtQ) | 2.118 | 0.0066 |
| (ic vs ip) x (GtE vs GtQ) | 0.1970 | 0.3221 |
| tissue x (ic vs ip) x (GtE vs GtQ) | 0.3751 | 0.3931 |

| **ANOVA table** | **SS (Type III)** | **DF** | **MS** | **F (DFn, DFd)** | **P value** |
| --- | --- | --- | --- | --- | --- |
| tissue | 9353 | 2 | 4676 | F (2, 86) = 14.68 | <0.0001 |
| (ic vs ip) | 7863 | 1 | 7863 | F (1, 86) = 24.68 | <0.0001 |
| (GtE vs GtQ) | 104802 | 1 | 104802 | F (1, 86) = 329.0 | <0.0001 |
| tissue x (ic vs ip) | 3388 | 2 | 1694 | F (2, 86) = 5.317 | 0.0066 |
| tissue x (GtE vs GtQ) | 3395 | 2 | 1698 | F (2, 86) = 5.329 | 0.0066 |
| (ic vs ip) x (GtE vs GtQ) | 315.9 | 1 | 315.9 | F (1, 86) = 0.9916 | 0.3221 |
| tissue x (ic vs ip) x (GtE vs GtQ) | 601.5 | 2 | 300.7 | F (2, 86) = 0.9439 | 0.3931 |
| Residual | 27399 | 86 | 318.6 |  |  |
