# Supplementary material for: Distinct prion conformers from brain and peripheral tissues of gene-targeted mice produce convergent CWD strain properties
Source: PLoS Pathog. 2026 Jun 4;22(6):e1014303. doi: 10.1371/journal.ppat.1014303 (PMC13252839; doi:10.1371/journal.ppat.1014303)
Supplement: S4 Table — (DOCX) [file ppat.1014303.s016.docx]

| **Intraperitoneal** | | | | | | | | | | | | |
| --- | --- | --- | --- | --- | --- | --- | --- | --- | --- | --- | --- | --- |
| **Host** | **Brain inoculum** | | | | **Spleen inoculum** | | | | **Muscle inoculum** | | | |
|  | Rep 1 | Rep 2 | Rep 3 | **Mean** | Rep 1 | Rep 2 | Rep 3 | **Mean** | Rep 1 | Rep 2 | Rep 3 | **Mean** |
| **GtE** | 6.9 | 7.0 | 7.6 | **7.2** | 7.3 | 7.6 | 7.8 | **7.6** | 7.1 | 7.7 | 7.5 | **7.5** |
| **GtQ** | 7.2 | 7.0 | 7.3 | **7.2** | 7.8 | 7.1 | 7.4 | **7.4** | 7.2 | 7.2 | 7.3 | **7.2** |
|  | | | | | | | | | | | | |
| **Intracerebral** | | | | | | | | | | | | |
| **Host** | **Brain inoculum** | | | | **Spleen inoculum** | | | | **Muscle inoculum** | | | |
|  | Rep 1 | Rep 2 | Rep 3 | **Mean** | Rep 1 | Rep 2 | Rep 3 | **Mean** | Rep 1 | Rep 2 | Rep 3 | **Mean** |
| **GtE** | 8.1 | 7.9 | 8.6 | **8.2** | 8.3 | 8.3 | 8.0 | **8.2** | 8.1 | 8.1 | 8.3 | **8.2** |
| **GtQ** | 8.4 | 8.9 | 8.2 | **8.5** | 8.6 | 8.6 | 8.8 | **8.7** | 8.7 | 8.2 | 8.2 | **8.3** |
