# Supplementary material for: Distinct prion conformers from brain and peripheral tissues of gene-targeted mice produce convergent CWD strain properties
Source: PLoS Pathog. 2026 Jun 4;22(6):e1014303. doi: 10.1371/journal.ppat.1014303 (PMC13252839; doi:10.1371/journal.ppat.1014303)
Supplement: S5 Table — (DOCX) [file ppat.1014303.s017.docx]

| **Route** | **Brain** | | | | **Spleen** | | | | **Muscle** | | | |
| --- | --- | --- | --- | --- | --- | --- | --- | --- | --- | --- | --- | --- |
|  | Rep 1 | Rep 2 | Rep 3 | **Mean** | Rep 1 | Rep 2 | Rep 3 | **Mean** | Rep 1 | Rep 2 | Rep 3 | **Mean** |
| **ic** | 8.3 | 8.3 | 8.6 | **8.4** | 5.7 | 5.2 | 5.8 | **5.5** | 4.4 | 5.0 | 5.4 | **4.9** |
| **ip** | 7.6 | 7.2 | 7.5 | **7.4** | 6.0 | 5.2 | 5.6 | **5.6** | 6.0 | 5.5 | 5.4 | **5.7** |
| **po** | 7.2 | 7.5 | 7.0 | **7.3** | 6.1 | 5.8 | 5.6 | **5.9** | 6.0 | 4.7 | 5.0 | **5.2** |
