# Supplementary material for: Distinct prion conformers from brain and peripheral tissues of gene-targeted mice produce convergent CWD strain properties
Source: PLoS Pathog. 2026 Jun 4;22(6):e1014303. doi: 10.1371/journal.ppat.1014303 (PMC13252839; doi:10.1371/journal.ppat.1014303)
Supplement: Attachment — Submitted filename: Response to Reviewers- 2.docx [file ppat.1014303.s019.docx]

PPATHOGENS-D-26-00271

We thank the editor and the reviewers for their comments and suggestions to which we have responded in full, and which have significantly improved the quality and clarity of our manuscript. Here we describe how we have responded in the revised manuscript. The editor’s and the reviewers’ comments are in **black,** and our responses to their feedback are in **blue**.

The reviewers agreed that the paper was of interest and important for understanding the mechanisms underlying prion strains. However, there were concerns raised about some of the data. Two of the reviewers asked for clarification of the PrPSc glycosylation data in Figure 1, including the addition of data showing the PrPC glycopattern in the tissues analyzed. I would further suggest that it might be helpful to use endoglycosidases such as PNGaseF or EndoH to define the glycoforms being quantified, especially in the case of muscle where diglycosylated PrPSc is shown as underrepresented even though there there still appears to be 3 PrPSc bands present (Figure 1B). Other interpretation concerns raised by the reviewers that should be addressed regard the apparent pooling of incubation time data from two different mouse strains prior to statistical analysis (Figure 3B and C), the impact of sample titer on the data, and the histopathological identification of PrPSc in muscle (Figure 1G). Finally, there were also some concerns expressed about the clarity of presentation and lack of experimental details with regard to more precise definitions of clinical signs, sufficiently detailed figure legends, source of inocula, and number of transmission. Please clearly address all of these issues in the revised manuscript.

In the original submission, we included western blot analyses of PrP^C^ from brain, spleen, and muscle homogenates in Supplemental Figure 1, which showed a dose-response of PrP^C^ levels in these tissues. We conducted experiments in response to the editor’s comments. In these additional analyses we enzymatically removed N-linked glycans from PrP with PNGaseF and probed western blots with monoclonal antibodies PRC5 and SAF32. These two antibodies allowed us to distinguish full-length PrP^C^ from its N-glycosylated, proteolytically processed derivative, C1. We include these data in Figure 1 of our revision, and have revised the first paragraph of the results section as follows:

*GtE mice express elk PrP^C^ in the brain and peripheral tissues at levels equivalent to mouse PrP^C^ expressed in wild-type mice (11, 13). Western blotting demonstrated that PrP^C^ levels were the highest in the brain tissue, followed by spleen and muscle (Fig S1). This finding supports previous analyses in wild-type and knock-in mice and higher mammals (12, 24-27). In order to assess endoproteolytic processing of elk PrP^C^ in different tissues of GtE mice, we enzymatically removed N-linked glycans with PNGase F and probed Western blots with mAb PRC5 or SAF32 (Fig 1A-C). The epitopes of mAbs PRC5 and SAF32 reside in the globular domain and octapeptide repeats of PrP^C^, respectively. Accordingly, while PRC5 recognizes full-length PrP and C1 (28, 29), SAF32 recognizes only full-length PrP. Probing de-glycosylated PrP^C^ with SAF32 revealed that full-length PrP^C^ from brain and muscle migrated at ~ 27 kDa, while full-length PrP^C^ from spleen migrated at ~ 24 kDa. Also, while full-length PrP^C^ in brain and muscle was predominately fully glycosylated, full-length PrP^C^ from spleen was almost entirely un-glycosylated. These findings build upon previous exploratory analyses of PrP^C^ in different tissues (23-26). The presence of significant amounts of de-glycosylated C1 following PNGase F treatment also revealed that the majority of C1 in these tissues was N-glycosylated. These findings reveal that elk PrP^C^ is differentially glycosylated and processed in spleen, muscle, and brain tissues of GtE mice.*

Please see comments below directly addressing specific reviewer comments regarding ‘pooling of incubation time data’, ‘the impact of sample titer on the data’, and the ‘histopathological identification of PrPSc in muscle’. Additionally, please see our comments below and their corresponding clarifications/corrections to the manuscript.

**Reviewers' Comments:**

Reviewer's Responses to Questions

**Part I - Summary**
Please use this section to discuss strengths/weaknesses of study, novelty/significance, general execution and scholarship.

Reviewer #1: Dear Drs. DeFranco, et. al,
Your current manuscript expands the characterization of CWD-infection in two gene targeted mouse models that express cervid prion protein (GtE and GtQ) developed by the Telling laboratory. New to this manuscript is a detailed characterization and comparison of the biochemical and infectious properties of CWD prions present in peripheral tissues (spleen and muscle) compared to brain derived prions from CWD-infected GtE mice. The authors show that prions present in peripheral tissues have different biochemical and conformational properties compared to prions found in brains of the same mice. CWD-infected tissue homogenates (muscle, spleen and brain) from GtE were then inoculated either IP or IC to additional GtE mice. The resulting disease and prion properties were identical regardless of the tissue source of the prions, however the route of inoculation used (IP or IC) created different clinical outcomes and prions with differing properties. This later finding has been published previously (JID 2026). Overall, the current manuscript demonstrates how tissue specific factors may influence prion properties and expands the characterization of the Gt mouse model to confirm involvement of CWD infection in peripheral tissues, similar to what is found in CWD-infected cervids, adding additional support to this model system for CWD pathogenesis and transmission studies. I have listed a few areas of concern/confusion below, along with some minor typos and inconsistencies to address.

Reviewer #2: This excellent study by DeFranco et al. investigates three distinct routes of chronic wasting disease (CWD) prion transmission to cervidised Gt mice and explores how these routes influence prion strain outcomes. The authors show that clearly distinct prion strain properties emerge in different tissues (brain, spleen, and muscle) independently of the infection route. However, intracerebral (ic) inoculation results in markedly reduced prion accumulation in peripheral tissues compared with peripheral routes, while in all cases the brain contains by far the highest prion abundance.

A central finding is that the conformational properties of CWD prions appear to be shaped by the route of inoculation, but not by the tissue source of inoculum. This is an important result that advances our understanding of prion strain or sub-strain selection mechanisms.

The authors further demonstrate the dominating influence of the environment on prion strain convergence using the RK cell culture model.

There is also an interesting demonstration of a component in the muscle tissues that appears to inhibit prion propagation - this is an intriguing and potentially significant observation.

The manuscript presents a large body of well-executed experiments with clear reasoning, robust data supporting each conclusion, and thoughtful discussion. I strongly support publication of this work in PLOS Pathogens, where it will undoubtedly be of great interest to the prion research community.

Reviewer #3: The manuscript by de Franco and colleagues describes a deep biochemical analysis of PrPSc in brain, lymphoid tissue, and muscle of CWD passaged in cervid PrPC expressing knockin mice. Differences were observed among infected tissues biochemically, yet upon transmission to mice intracerebrally or intraperitoneally, the strains converged to show the same conformational properties within a common inoculation route. Overall the novelty of the manuscript lies in the deep dive into the conformational properties of Gt mouse-adapted CWD prions from different organs that converge into a single strain upon injection by a common route, yet differ between the routes used, indicating that the route, and not the organ, is the essential determinant. This study is fundamentally important for understanding prion disease strains. Most concerns with this report revolve around the need for further clarification of the experimental paradigm and conclusions.

**Part II – Major Issues: Key Experiments Required for Acceptance**
Please use this section to detail the key new experiments or modifications of existing experiments that should be absolutely required to validate study conclusions.

Generally, there should be no more than 3 such required experiments or major modifications for a "Major Revision" recommendation. If more than 3 experiments are necessary to validate the study conclusions, then you are encouraged to recommend "Reject".

Reviewer #1: 1. Figure 3B&C/Table 1/Associated text and figure legends.
It appears that the primary goal of the data included in Fig3 B&C and Table 1 was to show that incubation periods were similar among mice inoculated with the three different tissue types. This is currently very difficult to pull from the presentation of the data. Were the incubation periods for the GtE and GtQ mice summed within each column in Figs 3B & C for this analysis? The box and whisker plots make it seem like they were… This seems very unfair statistically given that the previous papers and current manuscript clearly show that GtQ mice are very delayed compared to GtE mice. Pooling mice among two strains with clearly different incubation periods will alter any statistical test that is mathematically based on ranges, SEM or SD. These two strains of Gt mice should be looked at independent of each other, not pooled. I suspect this will also change the conclusion of statistical among the IC inoculated GtE mice. The legend (or methods) should also clearly state how the groups were compared to reach an “ns” conclusion across the board. Additionally, the design of table 1 and statistical comparisons seems to emphasize the difference between GtE and GtQ mice, yet this does not seem to be a goal of the current manuscript and only adds confusion.

We have separated the incubation time data as requested. The data are now shown in revised graphs in Figure 3B and 3C. We also conducted a three-way ANOVA analysis to determine the effects of the route of inoculation, tissue type used as inoculum, and the genotype of the mouse model (Table S3 and supporting data file). These analyses demonstrate that incubation period variance caused by the inoculation route and tissue type used as inoculum were marginal, while genotype had a profound effect. We have amended the results section to address these new analyses:

*Challenges of Gt mice with brain, spleen, and muscle tissue homogenates produced disease in all cases (Fig 3B-E, Table 1). Average times to disease onset ranged from 276 to 306 d in ic-challenged GtE mice and from 346 to 376 d in ic-challenged GtQ mice. Mean incubation periods ranged from 297 to 320 d in ip-challenged GtE mice and from 359 to 400 d in ip-challenged GtQ mice. Three-way ANOVA showed only modest influences of the route of inoculation and whether inocula originated from brain, spleen or muscle, but a significant effect of host genotype (Table S3). Indeed, in accordance with our previous observations following transmissions of natural CWD isolates from North America and South Korea (11, 13, 15, 45, 46), in all cases, disease was significantly more rapid in GtE than GtQ mice (Table 1).*

2. Methods on clinical signs. While this reviewer appreciates the data shown in Fig 3D, there needs to be more clarification on some of the clinical signs described. For example, what was different between truncal ataxia and impaired balance? Ataxia can lead to perceived issues with balance, and these can be very difficult to differentiate from ataxia with a visual assessment of a mouse. What was meant by a modified gait? Why wasn’t a mouse with a flattened gait also scored as a modified gait? In figure 3D the gait scores seemed mutually exclusive of each other, so more description of how they differed would be helpful. (add to methods)

We have added clarifying statements in the methods section.

*For clarity, truncal ataxia refers to any clinical sign encompassing loss of coordination, loss of grip strength, impaired balance while the mouse is stationary or walking, and other gait abnormalities that are not related to balance such as flattened gait. Impaired balance is assessed while the mouse is stationary or walking. Flattened gait is characterized by a depressed and lowered posture of the mouse while walking. Modified gait is identified by any condition that results in the limitation – usually in the hindlimbs – or inability of the mouse to walk.*

3. Discussion (lines 408-415). I would ask that the authors consider an entirely opposite conclusion to the one citing a low dilution of infectious material as the explanation for similar incubation periods. In most prion models, plateaus are observed with the most concentrated (high titer) inoculum (essentially the model cannot go any faster). As the infectious dose decreases, incubation periods spread out and time increases. None of your incubation period data has the appearance of “spreading out”, nor do you have any evidence that dose of prions was low in brain and spleen. The tissue homogenates of brain and spleen derived from clinically ill animals used for your inoculation were strongly positive for PrPSc by many of your assays and undoubtedly harbored a very high titer of infectivity at 1%, not a low level. Unfortunately, in the absence of any titration data in the gene targeted mice it is very difficult to speculate on why the incubation periods are as similar as they are in this mouse model. Future studies with these mice should include some end-point titrations of CWD inoculum by both IP and IC routes to help understand the dose-incubation time relationships in the Gt mice.

In hindsight, we agree with the reviewer’s comment, and apologize for the confusion generated by our speculative conclusions. We have modified and moved these discussion points to the previous paragraph:

*Moreover, compared with peripheral tissues, diseased brain tissue harbors higher levels of PK-resistant PrP^Sc^, greater in vitro amplification efficiency by PMCA, increased RT-QuIC seeding activity, and higher infectious titers upon infection of RK-E cells. Despite these differences, brain, spleen, and muscle homogenates produced comparable incubation times in GtE and GtQ mice. Further investigations, for example using endpoint titrations of CWD prions from brain and peripheral tissues, may provide insights into the basis of this discrepancy.*

Reviewer #2: N/A

Reviewer #3: Does PrPC influence the PrPSc glycopattern? A blot showing PrPC properties in brain, spleen, and muscle would be important to show how the glycoform pattern of PrPSc compares with PrPC sizes and glycoform profile in the spleen and muscle as compared to brain of Gt mice.

Please see the previous comment regarding the additional PrP^C^ glycoform analyses.

Fig. 1G: The histology in panel shows PrPSc in a peripheral nerve with myelinated axons, not myocytes. Was there also PrPSc in myocytes by IHC? Perhaps the PrPSc signal in muscle tissue is derived from peripheral nerve bundles?

We have added a supplemental figure showing the difference between muscle fibers and muscle spindles. As previously reported (citation 37), in which they determine the different between muscle fibers and muscle spindles based on tissue morphology, we also find that PrP^Sc^ deposition is in the muscle spindles. We also include IHC analyses of uninfected and KO muscle tissues, which were conducted during the same experiment as the infected samples in Figure 1, in the new supplemental figure. We have amended the sentence in the results section in which we discuss these data:

*Immunohistochemical analyses also confirmed CWD prion accumulation in muscle spindles of GtE mice, which is consistent with previous reports (37) (Fig 1J, Fig S5).*

**Part III – Minor Issues: Editorial and Data Presentation Modifications**
Please use this section for editorial suggestions as well as relatively minor modifications of existing data that would enhance clarity.

Reviewer #1: Line 18: “Infectious host range” as a keyword appears to be residual from a previous 2025 publication. The current manuscript does not include information regarding inter-species transmission.

We have removed this keyword and added ‘pathogenesis’ and ‘peripheral tissues’

Line 103: Consider being more specific here regarding the cell type. (Elk instead of mammalian)

We have modified the line to include ‘elk’:

*Here, we investigated the properties of prions in the brains and peripheral tissues of CWD-infected Gt mice and monitored the resulting strain properties following ic and intraperitoneal (ip) challenges in additional GtE and GtQ mice, as well as infection of susceptible cultured mammalian cells expressing elk PrP.*

Fig 1G. The IHC staining in the muscle tissue appears to be in the connective tissue between two myocytes, rather than in an actual myocyte. Consider including this detail. Figure 1G would also benefit from some support of a naïve muscle control, and/or a no primary antibody control, as the PrP staining does not look very convincing of typical PrPSc. Perhaps all of these images side by side warrant an additional supplemental panel.

Please see the above comment. We have included ‘naïve muscle controls’ from *Prnp*^-/-^ and uninfected GtE mice in the new Figure S5.

Lines 157-159: No GdnHCl data is provided for muscle. Was this assay performed or were levels of PrPSc in muscle insufficient for the analysis?

This is correct. We made several attempts to conduct conformational analyses using GdnHCl in which we pooled muscle tissues, and these experiments did not yield publishable data.

Line 353: typo, spleen instead of splenic

We have corrected this inaccuracy:

*We observed that the localization of CWD prions in the spleen and skeletal muscles of diseased GtE and GtQ mice is reminiscent of previous findings of lab-adapted mouse prion strains in wild-type mice*

Line 439 prion, not prions

We have corrected this inaccuracy:

*Because the study reported here was successful in understanding the properties of North American CWD prions, it warrants further investigation of other prion strains in peripheral tissues. Because of the diversity and instability of CWD strains in Northern Europe (44, 77, 78)…*

Lines 480-482 and line 605 are not consistent regarding the necropsy methods for preparing the brain for IHC. (coronal vs sagittal)

While we bisect some brains down the midline, we analyzed coronal sections for all brains tissues. We have added a clarifying sentence:

*We processed coronal sections for all formalin-fixed brain tissues.*

Line 538: Please provide your current source of D18 antibody

We have added this clarifying sentence:

*The D18 antibody was produced in-house from cultured Chinese hamster ovary (CHO) cells provided by Richard Bessen (Colorado State University, Colorado, USA) (77).*

Reviewer #2: Minor points:

1. It is understood that glycoform ratios quantified in Fig. 1C are an average of all routes of infection, could that be clearly stated also in the fig legend?

This is correct. We have added clarifying wording to the figure legend.

***E.****, ratio of CWD PrP^Sc^ glycoforms from diseased GtE brain, spleen, and muscle tissue following all routes of inoculation from Fig 1F. Data points represent the mean (±SD) of the relative proportion from three animals of di-glycosylated (red), mono-glycosylated (blue), and un-glycosylated (gold) CWD PrP^Sc^. Locations of glycoform quantifications are described in Fig S2.*

2. In Fig. 1C the glycoform ratios are from somewhat overexposed WBs, several exposures would be helpful if available for a supplementary figure, but not critical.

We have included additional images of the blot taken at different exposures in Figure S2.

3. Fig. 1H legend seems to be lacking some information, it only mentions ip inoculation, however the lanes are labeled ic as well and it is not clear how much material was loaded in each lane.

We have provided clarity to the figure legend:

*Tissues were collected from uninfected or diseased GtE mouse following ic or ip inoculation with elk CWD prions. For PK-treated samples, 50 µg of protein was loaded. For NaPTA precipitated samples, 100 µg of protein was loaded for brain samples, 2 mg for spleen samples, and 4 mg for muscle samples.*

4. Lines 671 - 673 Fig 1 legend error, should probably be:
"D. – E. immunohistochemical analyses of spleen tissue from an ip-inoculated diseased GtE mouse; D. was probed with anti-PrP Fab d18;
E. was probed with anti-follicular dendritic cell (FDC) Ab (clone FDC-M1) or anti-CD21/CD35 ab (clone 7G6) as indicated."

We have changed the figure panels, and these are reflected in the legend:

***G. – H.****, immunohistochemical analyses of serial spleen tissue sections from an ip-inoculated diseased GtE mouse;* ***G.,*** *was probed with anti-PrP Fab d18;* ***H.,*** *was probed with anti-follicular dendritic cell (FDC) Ab (clone FDC-M1) or anti-CD21/CD35 ab (clone 7G6) as indicated.*

5. Not sure how PrP was mapped onto the cell type in spleen.

As done previously (citation 23), we confirmed the localization of germinal centers in GtE mice using FDC and CD21/CD35 antibodies. We have modified the text to state that we observed prion-specific staining in the germinal centers of ip-inoculated GtE splenic tissue and removed the ‘mapping’ onto a specific cell type.

*We probed with a follicular dendritic cell (FDC)-specific marker, FDC-M1 and a CD21/CD35 antibody to confirm the location of germinal centers in GtE mice (Fig 1H; Fig S4). We observed anti-PrP immunostaining in the germinal centers of ip-inoculated GtE splenic tissue (Fig 1G; Fig S3), which is consistent with previous findings from CWD-infected cervids (30) and laboratory-adapted rodent prions (31-34).*

6. In Fig. 2 the "I." seems to be missing

We apologize for this confusion. We have removed I. in the figure legend for clarity:

***H.****, quantification (±SD) for PMCA experiments for muscle and brain seeds. Reactions with 10 % (w/v) of KO muscle homogenate subjected to PMCA are purple, reactions with 10 % (w/v) of KO brain homogenate subjected to PMCA are black, and unamplified seeds in PMCA are gray.*

7. Fig. 4 legend for panels A-F seems to incorrectly report brain vs. muscle homogenate challenge.
We apologize for this confusion. We have made this correction and modified the figure legend to improve clarity:

*Prions from the brains of Gt mice following* ***A.****, intraperitoneal (ip) inoculation with CWD-infected brain homogenate;* ***B.****, ip inoculation with CWD-infected spleen homogenate;* ***C.****, ip inoculation with CWD-infected muscle homogenate;* ***D.****, intracerebral (ic) inoculation with CWD-infected brain homogenate;* ***E.****, ic inoculation with CWD-infected spleen homogenate;* ***F.****, ic inoculation with CWD-infected muscle homogenate.*

8. The text does not always specify all the details of the experiment, for example, the route of inoculation:
-lines 321-323: "These conformational stability analyses mirror our findings of the conformational properties of CNS prions from GtE and GtQ mice following inoculation with either brain, spleen, or
muscle tissues (Fig 4G)"

We have modified the sentence to improve clarity:

*These indistinguishable conformational properties mirror our CSA experiments using CNS prions, in which the [GdnHCl_1/2_] values were equivalent following transmission brain, spleen, and muscle tissues following either ip or ic inoculation (Fig 4G).*

-lines 408-411: "Despite prion titer differences of
several orders of magnitude between brain, spleen, and muscle tissues, these inoculum sources unexpectedly produced no differences in time to disease onset following transmission to GtE and GtQ mice"

We have revised this section of the text per a review’s comment. Please see above.

9. line 518: Tris-HCl?

This is correct. We have modified the text:

*For western blots in Fig 7E; Fig S11B, cultured cell monolayers were harvested using mechanical dissociation with cold lysis buffer (50 mM Tris-HCl, pH 8.0, 150 mM NaCl, 0.5% sodium deoxycholate, 0.5% Igepal CA-630).*

10. line 561: use "to seed"

We have corrected this inaccuracy:

*Protein misfolding cyclic amplification (PMCA). Infected GtE tissue homogenates were used to seed PMCA reactions.*

11. line 702: "Table S4.2" - should likely read Table S4

This is correct. We have modified the text:

*For each group, three animals were analyzed, and the individual titer values are summarized in Table S2.*

Reviewer #3: The manuscript needs to include the species being studied – Gt mouse-adapted CWD prions; to clarify that the study is not peripheral organs from cervid CWD.
We have modified the title to reflect this suggestion:

*Distinct prion conformers from brain and peripheral tissues of gene-targeted mice produce convergent CWD strain properties*

-Abstract - Line 30: Add species here
Abstract: lines 35-38: It is unclear if the further transmissions that produced convergent properties were following intracerebral inoculation?
Line 41: first time route is mentioned in the Abstract. Adding the routes used to the Abstract would help justify the conclusion that the route is important. Since ic and ip strains did not converge, would be helpful to distinguish in the abstract, as the line on convergence was observed only when the same route was used.

We have modified the abstract text.

*Prions are unique infectious agents because different conformational properties of their constituent proteins are responsible for the manifestation of distinct strains. While prion replication in humans with sporadic Creutzfeldt-Jakob disease and cattle with bovine spongiform encephalopathy is primarily limited to the central nervous system (CNS), a seminal feature of chronic wasting disease (CWD) in cervids is high infectious titers in peripheral tissues, including skeletal muscles and the lymphoreticular system. Although extensive studies have assessed prion properties in the CNS, our understanding of prions in peripheral tissues remains limited. Here, we compared the strain properties of CWD prions in peripheral and CNS tissues of gene-targeted (Gt) mice. Our studies reveal differences in the biochemical and conformational properties of CWD prions, as well as the prion levels, between peripheral and CNS tissues. While this finding suggested that these tissues harbored distinct CWD prions, transmissions of muscle, spleen, and brain homogenates to Gt mice by the intraperitoneal route produced convergent strain properties. Importantly, transmission of these tissues by the intracerebral route resulted in different disease phenotypes than intraperitoneal inoculations. Additionally, while prion infection of CWD-susceptible cells revealed different titers in muscle, spleen, and brain tissues, the conformational properties of the resulting de novo prions were indistinguishable. While our findings support a role for tissue-specific cofactors that affect the biochemical and conformational properties of prions, they also show that these parameters do not solely dictate disease outcomes and that additional factors, particularly the route of inoculation, exert a more pronounced influence on strain outcomes.*


Adding more specifics to the Results described in the text would be helpful. What were the sizes of the truncated PrPSc and spleen and muscle as compared to brain? (line 148)

We have amended the text to include approximate molecular weights of the brain and spleen prions in the western blot in Fig. 1H:

*The molecular weight of splenic prions following NaPTA precipitation corresponded to truncated PrP of PK-resistant PrP^Sc^ from brain tissue, which was between 17-27 kDa. The molecular weight of purified brain prions was similar to full-length PrP at 25-35 kDa (Fig 1H; Fig S5).*

CWD derived from muscle lacked high levels of diglycosylated PrPSc (Line 140). What glycoform pattern the muscle did show?

Please see previous comment regarding the addition of analyses of PrP^C^ in the muscle.

Line 159. It would be helpful to note the differences in half maximal denaturation in brain versus spleen here.

We have modified the text to include the exact GdnHCl concentrations required to produce half maximal denaturation for brain and spleen prions.

*Prions from brain tissue required elevated levels of GdnHCl of 2.01 M to produce half-maximal denaturation ([GdnHCl_1/2_]) compared to prions derived from spleen tissue which only required 1.20 M (Fig 1J).*

The finding of 10-fold higher levels of seeding activity in the brain of IC versus IP the inoculated mice is interesting. The brainstem (medulla oblongata) seems to be a point of convergence as it had similarly high vacuolation scores by all routes and tissue inoculated. Was the level of PrPSc in the brainstem also similar between the routes?

We have conducted western blots published in DeFranco et al. *PNAS*, 2024 (Supplemental Figure 4), which showed similar levels of PrP^Sc^ in the brainstem of ip and ic inoculated mice.

Line 245. Listing the forebrain structures referred to here would be helpful.

We have modified the text to include specific brain regions:

*While the location of spongiform degeneration of ip-inoculated GtE and GtQ mice was invariable, with higher severity in the hindbrain, ic-inoculated mice harbored higher levels of vacuolation and modest differences between GtE and GtQ mice in the superior colliculus, thalamus, hippocampus, and septal area (Fig 5A-F).*

Line 248 describes CWD prions in IP inoculated mice being limited in the cortex and thalamus, and notes a difference as compared to the IC inoculated mice – what was the difference? Please describe the localization in the IC mice for comparison.
Line 249, what was the morphology of the aggregates after IP inoculation?

The IHC analyses in this figure were restricted to the cortex and thalamus regions. We have modified the text of these sections to provide more clarity:

*Immunohistochemical analyses of disease-associated PrP demonstrated that the cortex and thalamic regions of ip-inoculated GtE and GtQ mice contained limited CWD prion deposition composed of coarse aggregates (Fig 5G-I). In contrast, the brains of ic-inoculated GtE and GtQ mice contained high levels of PrP^Sc^ deposition in these regions, which comprised a mix of coarse and punctate aggregates (Fig 5J-L).*

Line 251. There were no discernible differences in PrPSc deposition between GT mice inoculated with brain, spleen, or muscle tissue. Does this refer to the location of the deposits or the morphology of the deposits, or both?

This refers to both. The text has been amended to include these specifics:

*There were no discernible differences in the location or morphology of prion deposition in diseased Gt mice inoculated with brain, spleen, or muscle tissues.*

Line 360 and 397, what aspect of prion strain properties were previously found to differ depending on the route? It would be helpful to add the results from previous study here for context.

We added additional information about our previous study:

*Our previous studies showed that following transmission of CWD prions from native cervids, the route of inoculation alters the conformational and biochemical properties of prions in the brain, which leads to different neuropathological phenotypes and levels of prion replication in the CNS (15). Additionally, we observed accelerated incubation periods upon serial passage of only ic inoculated mice, indicating that the properties of native CWD are modified following direct prion injection into the brain.*

How many transmissions were done in the serial transmissions? Please add to Results and Methods.

We added specifics about the serial transmissions to the Methods.

*Twelve serial transmissions were conducted using infected brain, spleen, or muscle homogenates diluted to 1 % (w/v). Four transmissions for each brain, spleen, and muscle tissues were conducted using GtE or GtQ mice by either the intracerebral or intraperitoneal routes as described above.*

Thank you for submitting your paper, "Distinct prion conformers from brain and peripheral tissues of gene-targeted mice produce convergent CWD strain properties", to PLOS Pathogens. We have checked over your submission and, before we can assign your manuscript to the Editor, we ask that you first please make the following modifications:

1) We have amended your Competing Interest statement to comply with journal style. We kindly ask that you double check the statement and let us know if anything is incorrect.

The amended Competing Interest statement is acceptable.

2) We do not publish any copyright or trademark symbols that usually accompany proprietary names, eg ©,  ®, or TM  (e.g. next to drug or reagent names). Therefore please remove all instances of trademark/copyright symbols throughout the text, including: Scientific™, Studio™, (cOmplete™, Bio-rad™, Studio™, SuperBlock™, Scientific™), IRDye®, and Immunospot® on the manuscript file.

We have removed all copyright or trademark symbols.

3) Thank you for stating "All animal work was performed in an AAALAC-accredited facility in compliance with the Guide for the Care and Use of Laboratory Animals. All procedures used in this study were performed in compliance with the Colorado State University Institutional Animal Care and Use Committee."

Please include the approval number(s), or a statement that approval was granted by the named board(s).

We included the internal code for Breeding number (5405) and Study number (4710) in the material and methods section.
4) We have noticed that you have uploaded Supporting Information files, but you have not included a list of legends. Please add a full list of legends for your Supporting Information files after the references list.

We generated .tif files for each supplemental figure and individual files for all six supplemental tables. We included a full list of legends in a separate file.

5) Please amend your detailed Financial Disclosure statement. This is published with the article, therefore should be completed in full sentences and contain the exact wording you wish to be published.
i). State what role the funders took in the study. If the funders had no role in your study, please state: “The funders had no role in study design, data collection and analysis, decision to publish, or preparation of the manuscript.”
ii). If any authors received a salary from any of your funders, please state which authors and which funders.

We have amended the Financial Disclosure statement. The authors did not receive a salary directly from the funders.
