# Supplementary material for: Distinct prion conformers from brain and peripheral tissues of gene-targeted mice produce convergent CWD strain properties
Source: PLoS Pathog. 2026 Jun 4;22(6):e1014303. doi: 10.1371/journal.ppat.1014303 (PMC13252839; doi:10.1371/journal.ppat.1014303)
Supplement: Peer review history

# Peer review: Decision Letter 0

**DOI:** 10.1371/journal.ppat.1014303.r001

**Authors:** Suzette Priola

24 Mar 2026

PPATHOGENS-D-26-00271

Distinct CWD prion conformers from different tissues produce convergent strain properties upon transmission to gene-targeted mice and cultured cells expressing cervid PrP

PLOS Pathogens

Dear Dr. DeFranco,

Thank you for submitting your manuscript to PLOS Pathogens. After careful consideration, we feel that it has merit but does not fully meet PLOS Pathogens's publication criteria as it currently stands. Therefore, we invite you to submit a revised version of the manuscript that addresses the points raised during the review process.

The reviewers agreed that the paper was of interest and important for understanding the mechanisms underlying prion strains. However, there were concerns raised about some of the data. Two of the reviewers asked for clarification of the PrPSc glycosylation data in Figure 1, including the addition of data showing the PrPC glycopattern in the tissues analyzed. I would further suggest that it might be helpful to use endoglycosidases such as PNGaseF or EndoH to define the glycoforms being quantified, especially in the case of muscle where diglycosylated PrPSc is shown as underrepresented even though there there still appears to be 3 PrPSc bands present (Figure 1B). Other interpretation concerns raised by the reviewers that should be addressed regard the apparent pooling of incubation time data from two different mouse strains prior to statistical analysis (Figure 3B and C), the impact of sample titer on the data, and the histopathological identification of PrPSc in muscle (Figure 1G). Finally, there were also some concerns expressed about the clarity of presentation and lack of experimental details with regard to more precise definitions of clinical signs, sufficiently detailed figure legends, source of inocula, and number of transmission. Please clearly address all of these issues in the revised manuscript.

Please submit your revised manuscript by May 23 2026 11:59PM. If you will need more time than this to complete your revisions, please reply to this message or contact the journal office at plospathogens@plos.org. When you're ready to submit your revision, log on to https://www.editorialmanager.com/ppathogens/ and select the 'Submissions Needing Revision' folder to locate your manuscript file.

Please include the following items when submitting your revised manuscript:

* A letter that responds to each point raised by the editor and reviewer(s). You should upload this letter as a separate file labeled 'Response to Reviewers'. This file does not need to include responses to any formatting updates and technical items listed in the 'Journal Requirements' section below.

* A marked-up copy of your manuscript that highlights changes made to the original version. You should upload this as a separate file labeled 'Revised Manuscript with Track Changes'.

* An unmarked version of your revised paper without tracked changes. You should upload this as a separate file labeled 'Manuscript'.

If you would like to make changes to your financial disclosure, competing interests statement, or data availability statement, please make these updates within the submission form at the time of resubmission. Guidelines for resubmitting your figure files are available below the reviewer comments at the end of this letter.

We look forward to receiving your revised manuscript.

Kind regards,

Suzette A. Priola

Academic Editor

PLOS Pathogens

Neil Mabbott

Section Editor

PLOS Pathogens

Sumita Bhaduri-McIntosh

Editor-in-Chief

PLOS Pathogens

orcid.org/0000-0003-2946-9497

Michael Malim

Editor-in-Chief

PLOS Pathogens

orcid.org/0000-0002-7699-2064

**Journal Requirements:**

If the reviewer comments include a recommendation to cite specific previously published works, please review and evaluate these publications to determine whether they are relevant and should be cited. There is no requirement to cite these works unless the editor has indicated otherwise.

1) Please ensure that the CRediT author contributions listed for every co-author are completed accurately and in full.

At this stage, the following Authors/Authors require contributions: Jenna Crowell, and Glenn C Telling. Please ensure that the full contributions of each author are acknowledged in the "Add/Edit/Remove Authors" section of our submission form.

The list of CRediT author contributions may be found here: https://journals.plos.org/plospathogens/s/authorship#loc-author-contributions

2) We ask that a manuscript source file is provided at Revision. Please upload your manuscript file as a .doc, .docx, .rtf or .tex. If you are providing a .tex file, please upload it under the item type u2018LaTeX Source Fileu2019 and leave your .pdf version as the item type u2018Manuscriptu2019.

3) We do not publish any copyright or trademark symbols that usually accompany proprietary names, eg ©,  ®, or TM  (e.g. next to drug or reagent names). Therefore please remove all instances of trademark/copyright symbols throughout the text, including:

- ® on pages: 24, 27, and 28

- TM on pages: 24, 26, 27, and 28.

4) Please upload all main figures as separate Figure files in .tif or .eps format. For more information about how to convert and format your figure files please see our guidelines:

https://journals.plos.org/plospathogens/s/figures

5) We have noticed that you have uploaded Supporting Information files, but you have not included a list of legends. Please add a full list of legends for your Supporting Information files after the references list.

6) Please amend your detailed Financial Disclosure statement. This is published with the article. It must therefore be completed in full sentences and contain the exact wording you wish to be published.

1) State what role the funders took in the study. If the funders had no role in your study, please state: "The funders had no role in study design, data collection and analysis, decision to publish, or preparation of the manuscript."

2) If any authors received a salary from any of your funders, please state which authors and which funders..

If you did not receive any funding for this study, please simply state: u201cThe authors received no specific funding for this work.u201d

**Reviewers' Comments:**

Reviewer's Responses to Questions

**Part I - Summary**

Please use this section to discuss strengths/weaknesses of study, novelty/significance, general execution and scholarship.

Reviewer #1: Dear Drs. DeFranco, et. al,

Your current manuscript expands the characterization of CWD-infection in two gene targeted mouse models that express cervid prion protein (GtE and GtQ) developed by the Telling laboratory. New to this manuscript is a detailed characterization and comparison of the biochemical and infectious properties of CWD prions present in peripheral tissues (spleen and muscle) compared to brain derived prions from CWD-infected GtE mice. The authors show that prions present in peripheral tissues have different biochemical and conformational properties compared to prions found in brains of the same mice. CWD-infected tissue homogenates (muscle, spleen and brain) from GtE were then inoculated either IP or IC to additional GtE mice. The resulting disease and prion properties were identical regardless of the tissue source of the prions, however the route of inoculation used (IP or IC) created different clinical outcomes and prions with differing properties. This later finding has been published previously (JID 2026). Overall, the current manuscript demonstrates how tissue specific factors may influence prion properties and expands the characterization of the Gt mouse model to confirm involvement of CWD infection in peripheral tissues, similar to what is found in CWD-infected cervids, adding additional support to this model system for CWD pathogenesis and transmission studies. I have listed a few areas of concern/confusion below, along with some minor typos and inconsistencies to address.

Reviewer #2: This excellent study by DeFranco et al. investigates three distinct routes of chronic wasting disease (CWD) prion transmission to cervidised Gt mice and explores how these routes influence prion strain outcomes. The authors show that clearly distinct prion strain properties emerge in different tissues (brain, spleen, and muscle) independently of the infection route. However, intracerebral (ic) inoculation results in markedly reduced prion accumulation in peripheral tissues compared with peripheral routes, while in all cases the brain contains by far the highest prion abundance.

A central finding is that the conformational properties of CWD prions appear to be shaped by the route of inoculation, but not by the tissue source of inoculum. This is an important result that advances our understanding of prion strain or sub-strain selection mechanisms.

The authors further demonstrate the dominating influence of the environment on prion strain convergence using the RK cell culture model.

There is also an interesting demonstration of a component in the muscle tissues that appears to inhibit prion propagation - this is an intriguing and potentially significant observation.

The manuscript presents a large body of well-executed experiments with clear reasoning, robust data supporting each conclusion, and thoughtful discussion. I strongly support publication of this work in PLOS Pathogens, where it will undoubtedly be of great interest to the prion research community.

Reviewer #3: The manuscript by de Franco and colleagues describes a deep biochemical analysis of PrPSc in brain, lymphoid tissue, and muscle of CWD passaged in cervid PrPC expressing knockin mice. Differences were observed among infected tissues biochemically, yet upon transmission to mice intracerebrally or intraperitoneally, the strains converged to show the same conformational properties within a common inoculation route. Overall the novelty of the manuscript lies in the deep dive into the conformational properties of Gt mouse-adapted CWD prions from different organs that converge into a single strain upon injection by a common route, yet differ between the routes used, indicating that the route, and not the organ, is the essential determinant. This study is fundamentally important for understanding prion disease strains. Most concerns with this report revolve around the need for further clarification of the experimental paradigm and conclusions.

**********

**Part II – Major Issues: Key Experiments Required for Acceptance**

Please use this section to detail the key new experiments or modifications of existing experiments that should be absolutely required to validate study conclusions.

Generally, there should be no more than 3 such required experiments or major modifications for a "Major Revision" recommendation. If more than 3 experiments are necessary to validate the study conclusions, then you are encouraged to recommend "Reject".

Reviewer #1: 1. Figure 3B&C/Table 1/Associated text and figure legends.

It appears that the primary goal of the data included in Fig3 B&C and Table 1 was to show that incubation periods were similar among mice inoculated with the three different tissue types. This is currently very difficult to pull from the presentation of the data. Were the incubation periods for the GtE and GtQ mice summed within each column in Figs 3B & C for this analysis? The box and whisker plots make it seem like they were… This seems very unfair statistically given that the previous papers and current manuscript clearly show that GtQ mice are very delayed compared to GtE mice. Pooling mice among two strains with clearly different incubation periods will alter any statistical test that is mathematically based on ranges, SEM or SD. These two strains of Gt mice should be looked at independent of each other, not pooled. I suspect this will also change the conclusion of statistical among the IC inoculated GtE mice. The legend (or methods) should also clearly state how the groups were compared to reach an “ns” conclusion across the board. Additionally, the design of table 1 and statistical comparisons seems to emphasize the difference between GtE and GtQ mice, yet this does not seem to be a goal of the current manuscript and only adds confusion.

2. Methods on clinical signs. While this reviewer appreciates the data shown in Fig 3D, there needs to be more clarification on some of the clinical signs described. For example, what was different between truncal ataxia and impaired balance? Ataxia can lead to perceived issues with balance, and these can be very difficult to differentiate from ataxia with a visual assessment of a mouse. What was meant by a modified gait? Why wasn’t a mouse with a flattened gait also scored as a modified gait? In figure 3D the gait scores seemed mutually exclusive of each other, so more description of how they differed would be helpful. (add to methods)

3. Discussion (lines 408-415). I would ask that the authors consider an entirely opposite conclusion to the one citing a low dilution of infectious material as the explanation for similar incubation periods. In most prion models, plateaus are observed with the most concentrated (high titer) inoculum (essentially the model cannot go any faster). As the infectious dose decreases, incubation periods spread out and time increases. None of your incubation period data has the appearance of “spreading out”, nor do you have any evidence that dose of prions was low in brain and spleen. The tissue homogenates of brain and spleen derived from clinically ill animals used for your inoculation were strongly positive for PrPSc by many of your assays and undoubtedly harbored a very high titer of infectivity at 1%, not a low level. Unfortunately, in the absence of any titration data in the gene targeted mice it is very difficult to speculate on why the incubation periods are as similar as they are in this mouse model. Future studies with these mice should include some end-point titrations of CWD inoculum by both IP and IC routes to help understand the dose-incubation time relationships in the Gt mice.

Reviewer #2: N/A

Reviewer #3: Does PrPC influence the PrPSc glycopattern? A blot showing PrPC properties in brain, spleen, and muscle would be important to show how the glycoform pattern of PrPSc compares with PrPC sizes and glycoform profile in the spleen and muscle as compared to brain of Gt mice.

Fig. 1G: The histology in panel shows PrPSc in a peripheral nerve with myelinated axons, not myocytes. Was there also PrPSc in myocytes by IHC? Perhaps the PrPSc signal in muscle tissue is derived from peripheral nerve bundles?

**********

**Part III – Minor Issues: Editorial and Data Presentation Modifications**

Please use this section for editorial suggestions as well as relatively minor modifications of existing data that would enhance clarity.

Reviewer #1: Line 18: “Infectious host range” as a keyword appears to be residual from a previous 2025 publication. The current manuscript does not include information regarding inter-species transmission.

Line 103: Consider being more specific here regarding the cell type. (Elk instead of mammalian)

Fig 1G. The IHC staining in the muscle tissue appears to be in the connective tissue between two myocytes, rather than in an actual myocyte. Consider including this detail. Figure 1G would also benefit from some support of a naïve muscle control, and/or a no primary antibody control, as the PrP staining does not look very convincing of typical PrPSc. Perhaps all of these images side by side warrant an additional supplemental panel.

Lines 157-159: No GdnHCl data is provided for muscle. Was this assay performed or were levels of PrPSc in muscle insufficient for the analysis?

Line 353: typo, spleen instead of splenic

Line 439 prion, not prions

Lines 480-482 and line 605 are not consistent regarding the necropsy methods for preparing the brain for IHC. (coronal vs sagittal)

Line 538: Please provide your current source of D18 antibody

Reviewer #2: Minor points:

1. It is understood that glycoform ratios quantified in Fig. 1C are an average of all routes of infection, could that be clearly stated also in the fig legend?

2. In Fig. 1C the glycoform ratios are from somewhat overexposed WBs, several exposures would be helpful if available for a supplementary figure, but not critical.

3. Fig. 1H legend seems to be lacking some information, it only mentions ip inoculation, however the lanes are labeled ic as well and it is not clear how much material was loaded in each lane.

4. Lines 671 - 673 Fig 1 legend error, should probably be:

"D. – E. immunohistochemical analyses of spleen tissue from an ip-inoculated diseased GtE mouse; D. was probed with anti-PrP Fab d18;

E. was probed with anti-follicular dendritic cell (FDC) Ab (clone FDC-M1) or anti-CD21/CD35 ab (clone 7G6) as indicated."

5. Not sure how PrP was mapped onto the cell type in spleen.

6. In Fig. 2 the "I." seems to be missing

7. Fig. 4 legend for panels A-F seems to incorrectly report brain vs. muscle homogenate challenge.

8. The text does not always specify all the details of the experiment, for example, the route of inoculation:

-lines 321-323: "These conformational stability analyses mirror our findings of the conformational properties of CNS prions from GtE and GtQ mice following inoculation with either brain, spleen, or

muscle tissues (Fig 4G)"

-lines 408-411: "Despite prion titer differences of

several orders of magnitude between brain, spleen, and muscle tissues, these inoculum sources unexpectedly produced no differences in time to disease onset following transmission to GtE and GtQ mice"

It might be worth scanning the text to add these details where they're missing.

9. line 518: Tris-HCl?

10. line 561: use "to seed"

11. line 702: "Table S4.2" - should likely read Table S4

Reviewer #3: The manuscript needs to include the species being studied – Gt mouse-adapted CWD prions; to clarify that the study is not peripheral organs from cervid CWD.

- Title needs clarification of the species, ie Distinct mouse-adapted CWD prion conformers

-Abstract - Line 30: Add species here

Abstract: lines 35-38: It is unclear if the further transmissions that produced convergent properties were following intracerebral inoculation?

Line 41: first time route is mentioned in the Abstract. Adding the routes used to the Abstract would help justify the conclusion that the route is important. Since ic and ip strains did not converge, would be helpful to distinguish in the abstract, as the line on convergence was observed only when the same route was used.

Adding more specifics to the Results described in the text would be helpful. What were the sizes of the truncated PrPSc and spleen and muscle as compared to brain? (line 148)

CWD derived from muscle lacked high levels of diglycosylated PrPSc (Line 140). What glycoform pattern the muscle did show?

Line 159. It would be helpful to note the differences in half maximal denaturation in brain versus spleen here.

The finding of 10-fold higher levels of seeding activity in the brain of IC versus IP the inoculated mice is interesting. The brainstem (medulla oblongata) seems to be a point of convergence as it had similarly high vacuolation scores by all routes and tissue inoculated. Was the level of PrPSc in the brainstem also similar between the routes?

Line 245. Listing the forebrain structures referred to here would be helpful.

Line 248 describes CWD prions in IP inoculated mice being limited in the cortex and thalamus, and notes a difference as compared to the IC inoculated mice – what was the difference? Please describe the localization in the IC mice for comparison.

Line 249, what was the morphology of the aggregates after IP inoculation?

Line 251. There were no discernible differences in PrPSc deposition between GT mice inoculated with brain, spleen, or muscle tissue. Does this refer to the location of the deposits or the morphology of the deposits, or both?

Line 360 and 397, what aspect of prion strain properties were previously found to differ depending on the route? It would be helpful to add the results from previous study here for context.

How many transmissions were done in the serial transmissions? Please add to Results and Methods.

**********

PLOS authors have the option to publish the peer review history of their article (what does this mean?). If published, this will include your full peer review and any attached files.

If you choose “no”, your identity will remain anonymous but your review may still be made public.

**Do you want your identity to be public for this peer review?** For information about this choice, including consent withdrawal, please see our Privacy Policy.

Reviewer #1: No

Reviewer #2: No

Reviewer #3: No

**Figure resubmission:**

While revising your submission, we strongly recommend that you use PLOS’s NAAS tool (https://ngplosjournals.pagemajik.ai/artanalysis) to test your figure files. NAAS can convert your figure files to the TIFF file type and meet basic requirements (such as print size, resolution), or provide you with a report on issues that do not meet our requirements and that NAAS cannot fix.

After uploading your figures to PLOS’s NAAS tool - https://ngplosjournals.pagemajik.ai/artanalysis, NAAS will process the files provided and display the results in the "Uploaded Files" section of the page as the processing is complete. If the uploaded figures meet our requirements (or NAAS is able to fix the files to meet our requirements), the figure will be marked as "fixed" above. If NAAS is unable to fix the files, a red "failed" label will appear above. When NAAS has confirmed that the figure files meet our requirements, please download the file via the download option, and include these NAAS processed figure files when submitting your revised manuscript.

**Reproducibility:**

To enhance the reproducibility of your results, we recommend that authors of applicable studies deposit laboratory protocols in protocols.io, where a protocol can be assigned its own identifier (DOI) such that it can be cited independently in the future. Additionally, PLOS ONE offers an option to publish peer-reviewed clinical study protocols. Read more information on sharing protocols at https://plos.org/protocols?utm_medium=editorial-email&utm_source=authorletters&utm_campaign=protocols

# Author comment: Author response to Decision Letter 1

**DOI:** 10.1371/journal.ppat.1014303.r002

11 May 2026

Attachment Submitted filename: Response to Reviewers- 2.docx

# Peer review: Decision Letter 1

**DOI:** 10.1371/journal.ppat.1014303.r003

**Authors:** Suzette Priola

26 May 2026

Dear Pr. Telling,

We are pleased to inform you that your manuscript 'Distinct prion conformers from brain and peripheral tissues of gene-targeted mice produce convergent CWD strain properties' has been provisionally accepted for publication in PLOS Pathogens.

Before your manuscript can be formally accepted you will need to complete some formatting changes, which you will receive in a follow up email. A member of our team will be in touch with a set of requests.

Please note that your manuscript will not be scheduled for publication until you have made the required changes, so a swift response is appreciated.

IMPORTANT: The editorial review process is now complete. PLOS will only permit corrections to spelling, formatting or significant scientific errors from this point onwards. Requests for major changes, or any which affect the scientific understanding of your work, will cause delays to the publication date of your manuscript.

Should you, your institution's press office or the journal office choose to press release your paper, you will automatically be opted out of early publication. We ask that you notify us now if you or your institution is planning to press release the article. All press must be co-ordinated with PLOS.

Thank you again for supporting Open Access publishing; we are looking forward to publishing your work in PLOS Pathogens.

Best regards,

Suzette A. Priola

Academic Editor

PLOS Pathogens

Neil Mabbott

Section Editor

PLOS Pathogens

Sumita Bhaduri-McIntosh

Editor-in-Chief

PLOS Pathogens

orcid.org/0000-0003-2946-9497

Michael Malim

Editor-in-Chief

PLOS Pathogens

orcid.org/0000-0002-7699-2064

***********************************************************

Reviewer Comments (if any, and for reference):

Reviewer's Responses to Questions

**Part I - Summary**

Please use this section to discuss strengths/weaknesses of study, novelty/significance, general execution and scholarship.

Reviewer #1: The authors have made significant revisions to improve and clarify the manuscript. I have not further concerns.

Reviewer #2: All issues have been satisfactorily addressed.

Reviewer #3: The authors have addressed the major and minor issues with this manuscript, and have done excellent work to clarify the text.

**********

**Part II – Major Issues: Key Experiments Required for Acceptance**

Please use this section to detail the key new experiments or modifications of existing experiments that should be absolutely required to validate study conclusions.

Generally, there should be no more than 3 such required experiments or major modifications for a "Major Revision" recommendation. If more than 3 experiments are necessary to validate the study conclusions, then you are encouraged to recommend "Reject".

Reviewer #1: The authors have made significant revisions to improve and clarify the manuscript. I have not further concerns.

Reviewer #2: n/a

Reviewer #3: (No Response)

**********

**Part III – Minor Issues: Editorial and Data Presentation Modifications**

Please use this section for editorial suggestions as well as relatively minor modifications of existing data that would enhance clarity.

Reviewer #1: The authors have made significant revisions to improve and clarify the manuscript. I have not further concerns.

Reviewer #2: n/a

Reviewer #3: (No Response)

**********

PLOS authors have the option to publish the peer review history of their article (what does this mean?). If published, this will include your full peer review and any attached files.

If you choose “no”, your identity will remain anonymous but your review may still be made public.

**Do you want your identity to be public for this peer review?** For information about this choice, including consent withdrawal, please see our Privacy Policy.

Reviewer #1: No

Reviewer #2: No

Reviewer #3: No

# Editor report: Acceptance letter

**DOI:** 10.1371/journal.ppat.1014303.r004

**Authors:** Suzette Priola

Dear Dr. DeFranco,

We are delighted to inform you that your manuscript, "Distinct prion conformers from brain and peripheral tissues of gene-targeted mice produce convergent CWD strain properties," has been formally accepted for publication in PLOS Pathogens.

We have now passed your article onto the PLOS Production Department who will complete the rest of the pre-publication process. All authors will receive a confirmation email upon publication.

The corresponding author will soon be receiving a typeset proof for review, to ensure errors have not been introduced during production. Please review the PDF proof of your manuscript carefully, as this is the last chance to correct any scientific or type-setting errors. Please note that major changes, or those which affect the scientific understanding of the work, will likely cause delays to the publication date of your manuscript. Note: Proofs for Front Matter articles (Pearls, Reviews, Opinions, etc...) are generated on a different schedule and may not be made available as quickly.

Soon after your final files are uploaded, the early version of your manuscript, if you opted to have an early version of your article, will be published online. The date of the early version will be your article's publication date. The final article will be published to the same URL, and all versions of the paper will be accessible to readers.

For Research Articles, you will receive an invoice from PLOS for your publication fee after your manuscript has reached the completed accept phase. If you receive an email requesting payment before acceptance or for any other service, this may be a phishing scheme. Learn how to identify phishing emails and protect your accounts at https://explore.plos.org/phishing.

Thank you again for supporting open-access publishing; we are looking forward to publishing your work in PLOS Pathogens.

Best regards,

Sumita Bhaduri-McIntosh

Editor-in-Chief

PLOS Pathogens

orcid.org/0000-0003-2946-9497

Michael Malim

Editor-in-Chief

PLOS Pathogens

orcid.org/0000-0002-7699-2064
